# Blood immunophenotyping identifies distinct kidney histopathology and outcomes in patients with lupus nephritis

**DOI:** 10.1172/JCI181034

**Published:** 2025-06-19

**Authors:** Alice Horisberger, Alec Griffith, Joshua Keegan, Arnon Arazi, John Pulford, Ekaterina Murzin, Kaitlyn Howard, Brandon Hancock, Andrea Fava, Takanori Sasaki, Tusharkanti Ghosh, Jun Inamo, Rebecca Beuschel, Ye Cao, Katie Preisinger, Maria Gutierrez-Arcelus, Thomas M. Eisenhaure, Joel Guthridge, Paul J. Hoover, Maria Dall’Era, David Wofsy, Diane L. Kamen, Kenneth C. Kalunian, Richard Furie, Michael Belmont, Peter Izmirly, Robert Clancy, David Hildeman, E. Steve Woodle, William Apruzzese, Maureen A. McMahon, Jennifer Grossman, Jennifer L. Barnas, Fernanda Payan-Schober, Mariko Ishimori, Michael Weisman, Matthias Kretzler, Celine C. Berthier, Jeffrey B. Hodgin, Dawit S. Demeke, Chaim Putterman, Accelerating Medicines Partnership Rheumatoid Arthritis and Systemic Lupus Erythematosus (AMP RA/SLE) Network, Michael B. Brenner, Jennifer H. Anolik, Soumya Raychaudhuri, Nir Hacohen, Judith A. James, Anne Davidson, Michelle A. Petri, Jill P. Buyon, Betty Diamond, Fan Zhang, James A. Lederer, Deepak A. Rao

**Affiliations:** 1 Brigham and Women’s Hospital, Harvard Medical School, Boston, Massachusetts, USA.; 2 Lausanne University Hospital, University of Lausanne, Lausanne, Switzerland.; 3 The Feinstein Institutes for Medical Research, Northwell Health, Manhasset, New York, USA.; 4 Johns Hopkins University School of Medicine, Baltimore, Maryland, USA.; 5 School of Public Health and; 6 School of Medicine, University of Colorado, Anschutz Medical Campus, Aurora, Colorado, USA.; 7 New York University School of Medicine, New York, New York, USA.; 8 Boston Children’s Hospital, Harvard Medical School, Boston, Massachusetts, USA.; 9 Broad Institute of MIT and Harvard, Cambridge, Massachusetts, USA.; 10 Oklahoma Medical Research Foundation, Oklahoma City, Oklahoma, USA.; 11 University of California, San Francisco, San Francisco, California, USA.; 12 Medical University of South Carolina, Charleston, South Carolina, USA.; 13 University of California San Diego School of Medicine, La Jolla, California, USA.; 14 University of Cincinnati College of Medicine, Cincinnati, Ohio, USA.; 15 University of California, Los Angeles, Los Angeles, California, USA.; 16 University of Rochester Medical Center, Rochester, New York, USA.; 17 Texas Tech University Health Sciences Center, El Paso, Texas, USA.; 18 Cedars-Sinai Medical Center, Los Angeles, California, USA.; 19 University of Michigan, Ann Arbor, Michigan, USA.; 20 Albert Einstein College of Medicine and Montefiore Medical Center, Bronx, New York, USA.; 21 Azreili Faculty of Medicine, Zefat, Israel.; 22 The Accelerating Medicines Partnership RA/SLE Network is detailed in the Supplemental Acknowledgments.

**Keywords:** Autoimmunity, Immunology, Biomarkers, Lupus

## Abstract

Lupus nephritis (LN) is a frequent manifestation of systemic lupus erythematosus, and fewer than half of patients achieve complete renal response with standard immunosuppressants. Identifying noninvasive, blood-based immune alterations associated with renal injury could aid therapeutic decisions. Here, we used mass cytometry immunophenotyping of peripheral blood mononuclear cells in 145 patients with biopsy-proven LN and 40 healthy controls to evaluate the heterogeneity of immune activation and identify correlates of renal parameters. Unbiased analysis identified 3 immunologically distinct groups of patients that were associated with different patterns of histopathology, renal cell infiltrates, urine proteomic profiles, and treatment response at 1 year. Patients with enriched circulating granzyme B^+^ T cells showed more active disease and increased numbers of activated CD8^+^ T cells in the kidney, yet they had the highest likelihood of treatment response. A second group characterized by a high type I interferon signature had a lower likelihood of response to therapy, while a third group appeared immunologically inactive but with chronic renal injuries. The major immunologic axes of variation could be distilled down to 5 simple cytometric parameters that recapitulate several clinical associations, highlighting the potential for blood immunoprofiling to translate to clinically useful noninvasive metrics to assess immune-mediated disease in LN.

## Abstract 2

Blood immune profiling help stratify patients with lupus nephritis by kidney disease activity and outcomes, summarized through five key immune signatures

## Introduction

Lupus nephritis (LN) is a common and severe manifestation of systemic lupus erythematosus (SLE) that affects over 40% of patients (1) and substantially increases SLE-associated morbidity and mortality (2, 3). Advances in the understanding of renal inflammatory processes in patients with severe LN (4–8) have not yet translated into improved renal outcomes (9). Less than half of the patients treated with standard immunosuppressive regimens achieve complete renal response at 1 year, and 10% to 30% progress to end-stage renal disease (9–11). The heterogeneity of immune pathways involved in patients with SLE and LN contributes to the variability in treatment response and poses challenges in developing successful new therapies (12, 13).

Current stratification of patients with LN relies on histologic classification of the nature, intensity, and localization of glomerular involvement (International Society of Nephrology/Renal Pathology Society [ISN/RPS] class), as well as the degree of active inflammation and fibrosis in the glomeruli and tubulointerstitium (NIH activity and chronicity indices) (14). These features currently guide treatment decisions (15, 16) and inclusion criteria in clinical trials (class III, IV, and/or V). However they have several limitations: they do not inform regarding the inflammatory pathways involved, they have limited ability to predict long-term renal outcome, and they show discordance with clinical response (6, 17). Stratifying patients based on molecular or cellular features of immune-mediated pathways would improve the predictive value of kidney biopsy (18). Efforts to characterize inflammatory pathways involved in the kidney tissue in LN have identified signatures associated with poor clinical outcome, such as accumulation of CD4^–^ T cells (19), enriched fibrotic pathways, type I interferon (IFN-I) signaling (5), and specific proinflammatory cytokines and chemokines (6).

Alongside kidney biopsy analysis, identification of noninvasive methods to assess LN immune heterogeneity and activity could substantially improve disease management. Prior efforts to characterize immunologic diversity among patients with SLE, not restricted to LN, using blood transcriptomics and proteomics have identified distinct signatures. Evidence of upregulated IFN-I signaling as measured by IFN response gene signatures has been repeatedly identified in 50%–80% of patients with SLE and is associated with more severe disease, including renal manifestations (20, 21). Independently, a circulating neutrophil signature has been associated with active renal involvement in patients with SLE (22, 23) and appears to improve with renal response to treatment (24). More generally, SLE disease activity has been characterized by specific transcriptomic signatures involving myeloid, plasmablast (22, 23), B cell activation, and cell cycle pathways (23). Similarly, cytometric analyses have highlighted expanded activated B cells, plasmablasts, B helper T cells, and activated myeloid cells in the blood of patients with active SLE, including patients with LN (25–28). Clustering of patients with SLE based on blood immunophenotyping by flow cytometry has shown promise in identifying distinct groups of patients with SLE with different treatment responses (29). However, the extent to which these cellular signatures can inform renal pathology and treatment response across patients with LN is unclear.

Accordingly, this study was initiated to identify patterns of immune cell signatures in the blood of patients with LN that are associated with renal tissue activity and treatment response. By applying mass cytometry immunophenotyping to a prospective cohort of patients with biopsy-proven LN in a multiethnic/multiracial study across the United States, we have defined alterations in circulating T cells, B cells, myeloid cells, and NK cells. Classifications and unsupervised models revealed blood cellular profiles distinguishing 3 groups of patients that differed in renal activity and outcome. Paired single-cell RNA-Seq of kidney immune cells and urine proteomics identified further associations between these blood immunophenotype–defined groups, kidney infiltrates, and urine proteomic signatures.

## Results

### Overview of blood immunophenotyping in a longitudinal cohort of patients with active LN.

We analyzed peripheral blood mononuclear cells (PBMCs) by mass cytometry using four 48-marker panels in 145 patients with SLE and biopsy-proven LN class III, IV, and/or V, as well as 40 healthy controls enrolled in the Accelerating Medicines Partnership (AMP) SLE phase II study. Compared with the controls, patients with LN in this study were younger (median [IQR] age 33 [26–43] vs. 44 [27–60] years, *P* = 0.02) and more frequently female (87% vs. 70%, *P* = 0.02), Black (52% vs. 25%, *P* = 0.003), and Hispanic or Latino (31% vs. 10%, *P* = 0.008) (Supplemental Table 1; supplemental material available online with this article; https://doi.org/10.1172/JCI181034DS1). Within patients with LN, 68% had positive anti-dsDNA antibody and/or low serum complement levels, and 50% had extrarenal clinical manifestations of SLE at the time of biopsy. All patients had a clinical indication for renal biopsy based on proteinuria (defined as urine protein to creatinine ratio [UPCR] > 0.5 g/g) (30, 31). Most biopsies reported a proliferative classification (III or IV with or without V, 70%) compared with pure membranous (V, 30%). Sixty-nine percent of the patients had a history of previous renal biopsy; 61% had 1 or more prior episodes of biopsy-proven proliferative or membranous LN. Patients were treated following standard of care according to the judgment of their physician (30, 31). Clinical renal response was defined as previously reported in patients with a UPCR greater than or equal to 1 at baseline (32, 33). At 52 weeks, 28% achieved complete response, 24% a partial response, and 48% no response. Longitudinal blood samples from 50 patients were analyzed at week 12 and/or week 52 to determine the trajectory of immune cells following treatment.

**Figure 1:**
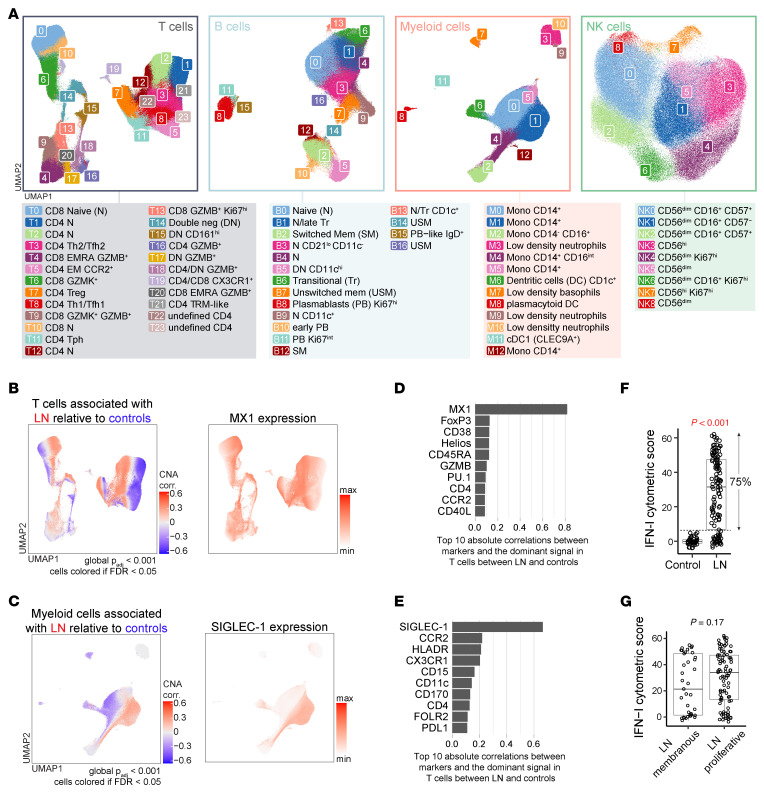
Blood immunophenotyping by mass cytometry captures the range of IFN-I signaling intensity in LN. (**A**) Cell type–specific clustering analysis for each panel (e.g., B cells in the B panel, including LN [*n* = 145] and controls [*n* = 40]). (**B**) T cell UMAPs of LN-associated cell neighborhoods adjusted for demographic factors (age, sex, ethnicity, and race) and false discovery rate (FDR < 0.05), along with MX1 expression. (**C**) Myeloid cell UMAPs of LN-associated cell neighborhoods adjusted for demographic factors and FDR (FDR < 0.05), along with Siglec-1 expression. (**D** and **E**) Top Spearman’s ρ absolute correlation between marker expression in the T panel (**D**) or myeloid panel (**E**) and the main axis of covarying neighborhood analysis (CNA) for T cells and myeloid cells, respectively. (**F**) Comparison of IFN-I cytometric scores between patients with LN (*n* = 125) and controls (*n* = 40) including samples stained with T, B, and myeloid panels; the dashed line represents 3 standard deviations above the control mean (mean + 3 SD = 6.46). (**G**) Comparison of IFN-I cytometric scores in patients with membranous LN (class V, *n* = 38) versus proliferative LN (class III or IV with or without V, *n* = 87). (**F** and **G**) Statistical significance was determined using Wilcoxon’s rank sum test.

After quality control, 267 PBMC samples were stained with panels to analyze T, B, myeloid, and NK cells (Supplemental Figure 1A and Supplemental Table 2), averaging approximately 111,000 cells per sample per panel (Supplemental Figure 1B). Batch effect correction reduced inter-batch variation without impairing cell type identification or LN versus control signal (Supplemental Figure 1, C–E). Using unsupervised clustering, we identified major cell types (T, B, myeloid, and NK) based on canonical markers and visualized them with uniform manifold approximation and projection (UMAP) (Supplemental Figure 1, F–H). These major cell lineages were then reclustered to define cell type–specific subsets (Figure 1A and Supplemental Figure 2). To validate the robustness of our unbiased analysis, we confirmed our ability to detect SLE-associated alterations in the frequency of rare cell populations, such as plasmablasts (CD20^–^CD19^int^CD27^hi^CD38^hi^) (27) and plasmacytoid dendritic cells (CD123^+^HLA-DR^+^IRF8^+^) (34) (Supplemental Figure 1H). Then, we confirmed an expansion of cell subsets previously associated with LN, including T peripheral helper (Tph) cells (cluster T11, CD4^+^PD-1^+^ICOS^+^CXCR5^–^) (27) and CD11c^+^ B cell subsets (clusters B5 and B9, CD11c^+^T-bet^+^CD21^–^IgD^+/–^) (25, 26, 28), independent of demographic variables (age, sex, ethnicity, and race) and immunosuppressive therapy (Supplemental Figure 1I). Broad differential abundance evaluation using covarying neighborhood analysis (CNA) (35) revealed that IFN-I–induced proteins (36), specifically MX1 in the T panel and Siglec-1 in the myeloid panel, accounted for the majority of the variance between patients with LN and controls across cell subsets, independent of immunosuppressive therapy (Figure 1, B–E, and Supplemental Figure 3A). This finding aligns with the well-established association of IFN-I with SLE (20–22) and supports our framework for capturing both signaling pathway activation and cell subset enrichments in this large mass cytometry dataset.

### IFN-I activation pathway does not correlate with LN class and renal activity.

Increased activity of IFN-I is associated with the severity of SLE, including LN, but its expression within patients with LN and its correlation with renal histology remain uncertain (5–7, 37–39). We defined a cytometric IFN-I signature combining the expression of MX1, Siglec-1, and ISG-15, a third IFN-I–induced marker (22, 23) included in the B cell panel that was strongly correlated with MX1 and Siglec-1 (MX1: *r* = 0.89, *P* < 0.001; Siglec-1: *r* = 0.75, *P* < 0.001) (Supplemental Figure 3, B and C). Seventy-five percent of LN patients in this cohort showed an increased IFN signature compared with controls (Figure 1F). IFN-I signaling was associated with the presence of anti-dsDNA and low complement (Supplemental Figure 3D), as reported in previous studies in SLE (37, 40, 41). However, no correlation was observed with proliferative class versus membranous class (Figure 1G), or renal activity by NIH index (Supplemental Figure 3, E and F), including the interstitial component, contrasting with the results from smaller cohorts (37, 39). In addition, no association was found with extrarenal clinical manifestations, baseline prednisone dose, or the use of immunosuppressants (Supplemental Figure 3E). These results indicate that IFN-I signaling was common across clinically and histologically heterogeneous patients with LN.

### Shifts in B, T, and myeloid circulating cells associate with histologic patterns of active LN.

**Figure 2:**
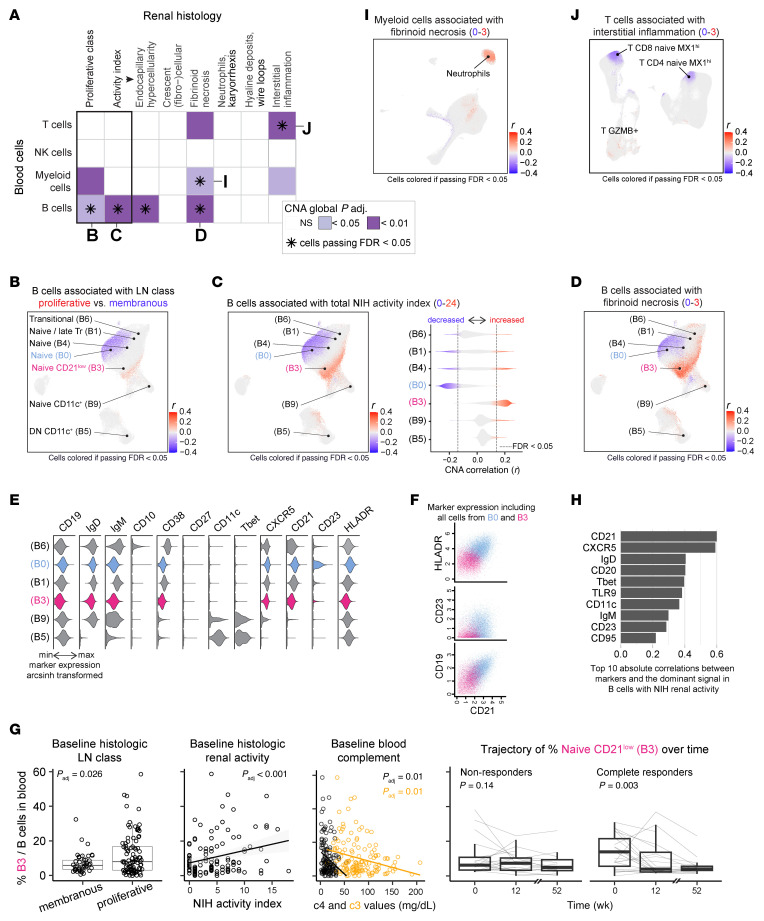
Association of circulating immune cell subsets with histologic patterns of active LN. (**A**) Heatmap showing associations between circulating blood cell types (*y* axis) and LN histologic patterns (*x* axis). CNA was used to test for associations, adjusting for demographic factors (age, sex, ethnicity, and race) and history of previous biopsy. Purple and light purple represent global adjusted CNA *P* values, with asterisks indicating significant local associations (FDR < 0.05). (**B**–**D**) B cell associations with LN histologic class (*n* = 124) (**B**), NIH renal activity index (*n* = 111) (**C**), and glomerular fibrinoid necrosis as defined by the NIH renal activity index (*n* = 90) (**D**); **C** includes a violin plot illustrating contributions of individual B cell clusters to the NIH activity index association. (**E**) Violin plots depicting selected protein expression levels in specific B cell clusters. (**F**) Scatterplot showing selected protein expression in B cell clusters B0 and B3. **E** and **F** include all subjects (LN = 145, controls = 40). (**G**) Proportion of B cell cluster B3 (percentage of total B cells) associated with LN histologic class (*n* = 140), renal activity index (*n* = 124), complement levels (*n* = 138), and longitudinal changes in non-responders (*n* = 19) versus complete responders (*n* = 23). Cross-sectional analyses used linear models, while longitudinal analyses used mixed-effects models with patients as a random effect. (**H**) Top correlations between B panel marker expression and the main axis of variation from the NIH activity index B cell association, using Spearman’s ρ correlations. (**I**) Association of myeloid cells with glomerular fibrinoid necrosis (*n* = 78). (**J**) Association of T cells with renal interstitial inflammation (*n* = 82). (**A**–**D**, **G**, **I**, and **J**) All cross-sectional statistical analyses shown were adjusted for demographic factors (age, sex, ethnicity, and race) and history of previous biopsy.

**Figure 3:**
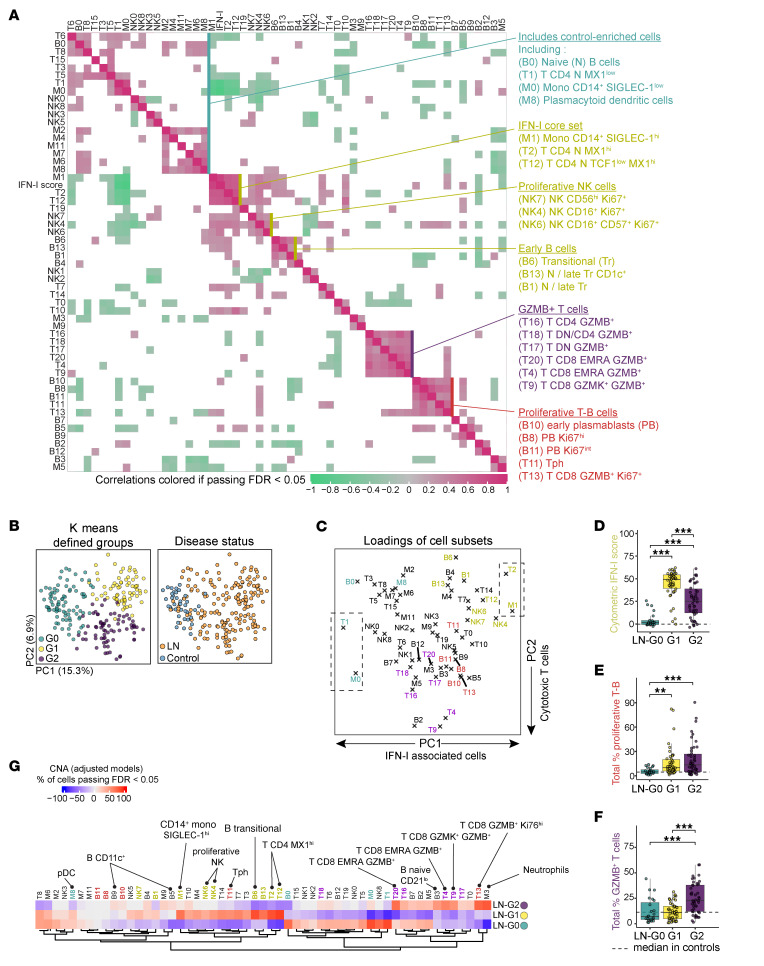
Distinct immune cell signatures in LN patients identified through unbiased blood immunophenotyping. (**A**) Correlation heatmap of 55 immune cell clusters and IFN-I cytometric signature in 115 LN patients, organized by hierarchical clustering. Spearman’s correlation coefficients are highlighted in color when correlations passed FDR < 0.05. (**B**) Principal component analysis (PCA) of 224 samples (39 controls, 115 baseline LN samples, 39 LN samples from week 12, and 31 LN samples from week 52) visualized by *K*-means–defined groups (left) and disease status (right). (**C**) Loadings of immune cell subsets on the first 2 PC dimensions, highlighting key cell groups associated with IFN-I and cytotoxic T cell signatures. (**D**–**F**) Comparisons of key immune cell signatures between each LN group at baseline (G0: *n* = 26; G1: *n* = 46; G2: *n* = 46). Statistical significance was determined using the Kruskal-Wallis tests with Dunn’s multiple comparisons. **P < 0.01, ***P < 0.001. (**G**) Heatmap showing covarying neighborhood associations of baseline immune cell subsets with LN groups relative to the 2 other groups. Colors represent the percentage of cells passing FDR < 0.05, as either enriched (red) or depleted (blue). (**A**–**G**) Selected immune cell subsets and groupings are consistently color-coded (green, yellow, purple, red) across all panels.

Since immunosuppressive treatment in SLE patients with LN is guided by the biopsy-defined proliferative class and the extent of renal activity (15, 16), we sought to identify immune alterations in blood that reflect histologic features in patients with LN. A comprehensive evaluation of each cell type revealed that both proliferative LN classes (III or IV with or without V) and renal activity were associated with shifts in B cell phenotypes (Figure 2A and Supplemental Figure 4, A and B), primarily within the naive B cells, even after adjusting for demographic features and history of previous biopsy (Figure 2, B and C), and when including only patients without immunosuppressants and with prednisone ≤ 5 mg at sampling (Supplemental Figure 4, C and D). Cells in cluster B3 (naive CD21^lo^) were the most positively associated with total renal activity score (Figure 2C), as well as specific features of glomerular endocapillary hypercellularity and fibrinoid necrosis (Figure 2D), while cells in cluster B0 (naive) were the most negatively associated (Figure 2, B and D). Cluster B3 appeared as naive CD21^lo^ B cells and was distinct from transitional cells (B6) and from activated CD11c^+^ subsets (B5, B9), lacking CD11c, T-bet, and CD10 and with intermediate CD38 expression (Figure 2E and Supplemental Figure 2). Cluster B3 cells also lacked CD23 and had lower CD19 and HLA-DR expression than cluster B0 cells (Figure 2, E and F). The proportion of naive CD21^lo^ B cells (B3) was associated with lower complement levels at baseline (e.g., C3 and C4), and decreased over time in patients with complete renal response at 52 weeks (Figure 2G). These clinical associations were reproducible when CD21 expression was examined in manually gated naive B cells (Supplemental Figure 5). Recent studies in B cells have described a similar heterogeneity among more differentiated double-negative (DN; CD27^–^IgD^–^) B cells, with the identification of DN3 cells (DN CD21^lo^CD11c^–^) suggested to be part of an extrafollicular response, similar to CD11c^+^ B cell subsets (42). In our study, CD21 expression on naive and DN B cells was more strongly associated with renal activity than was CD11c expression (Figure 3H).

Among other cell types, low-density neutrophils (M3; CD14^–^CD16^+^CD11b^+^CD15^hi^) (43) were strongly associated with glomerular fibrinoid necrosis (Figure 2, A and I, and Supplemental Figure 4, C and D). A subset of CD14^+^ monocytes (M5) expressing CD86^lo^ (Supplemental Figure 2), a protein downregulated with LPS stimulation and sepsis (44), was associated with NIH activity index and fibrinoid necrosis among patients without immunosuppression and with prednisone ≤ 5 mg (Supplemental Figure 4C), suggesting an inflammatory state. Shifts within the T cells correlated with the interstitial component of the NIH activity index but not with glomerular features (Figure 2, A and J).

### Cytotoxic T cells, proliferating T-B subsets, and IFN-I capture discrete immune axes in patients with LN.

These results highlighted relationships between blood immune cells and active renal injury. However, the limitations of renal activity to predict outcome, including in the AMP phase II cohort (32), and the immune heterogeneity observed in the cohort challenged us to approach the data in an unsupervised manner to identify broader cellular profiles associated with clinical features of LN. By examining 55 immune cell subsets and IFN-I score in patients with LN stained with 4 panels (*n* = 115), we observed sets of coordinated immune cell abundances (Figure 3A and Supplemental Figure 6A). Siglec-1^hi^ monocytes (M1) and MX1^hi^ naive CD4^+^ T cells (T2 and T12), marked by IFN-I proteins, formed a core set correlating with IFN-I score (Figure 3A). Other cells correlating and closely organized with the IFN-I core set included MX1^hi^ naive CD8^+^ T cells (T10), Ki67^hi^ NK cell subsets (NK4, NK6, NK7), transitional B cells (B6), and a small cluster of B cells with features of marginal zone precursors (B13; IgM^+^CD38^+^CD27^–^CD1c^+^) (45), consistent with the previously reported roles of IFN-I in NK cell activation (46) and in immature B cell expansion (47) (Figure 3A). Interestingly, T12 (TCD4 naive) was distinguished from T2 (TCD4 naive) by its reduced expression of TCF1, a key transcription factor in T cell stemness that is downregulated upon effector T cell differentiation (48) (Supplemental Figure 6, B and C). A similar pattern was observed in the TCD8 cells (T10 expressing low TCF1 compared with T0) (Supplemental Figure 6C). Using a published bulk RNA-Seq dataset (49), we found that IFN accelerated anti-CD3/CD28–induced downregulation of TCF7 in naive CD4^+^ T cells and, to a lesser extent, in naive CD8^+^ T cells (Supplemental Figure 6D). In contrast, MKI67 was not increased by IFN exposure (Supplemental Figure 6). In addition, in a single-cell RNA-Seq dataset of T cells from 7 patients with SLE treated with anifrolumab (anti-IFNAR), IFN-I blockade increased a T cell stemness score (TCF7, LEF1) (48) (Supplemental Figure 6, E and F). Together, these observations suggest that T12 cells represent naive T cells recently activated in a high–IFN-I environment.

We observed 2 other distinct sets of co-abundant immune cell clusters. One set included Tph cells (T11), activated memory B/early plasmablasts (B10), plasmablasts (B8, B11), and CD8^+^granzyme B^+^Ki67^+^ (T13) cells, comprising a set of T cells and B cells with a shared proliferative state (Ki67^+^), resembling subsets expanded in patients with newly diagnosed SLE (50) (Figure 3A). A second, distinct set of co-correlated T cells was unified by their expression of granzyme B, including CD8^+^ (T4, T9, T20), CD4^+^, and DN (T16, T17, T18) T cells (Figure 3A). The T13 CD8^+^granzyme B^+^Ki67^+^ cluster correlated with both the proliferative and the granzyme B^+^ cell sets and with IFN-I score (Figure 3A). However, non-proliferating granzyme B^+^ T cells did not correlate with IFN-I signature (Figure 3A), suggesting that an IFN signature and granzyme B expression represent two different axes of immune activation. Together, these results enabled visualization of distinct sets of highly correlated immune cell populations that are unified by specific functional features, providing a framework with which to interpret clinical associations of immune cell clusters in patients with LN.

### Blood immunophenotype identifies 3 groups of patients with LN.

We next asked whether the composition of circulating immune cell populations could help stratify patients with LN. We used a *K*-means clustering algorithm to group samples (baseline and longitudinal, *n* = 224) based on their similarities in the proportions of all blood immune cell subsets. This analysis identified 3 distinct groups of samples, which were clearly separated in both principal component analysis (PCA) and UMAP visualizations (Figure 3B and Supplemental Figure 7, A and B). When examining the composition of these groups, we found that group G0 included all control samples (*n* = 39) and a small subset of patients with LN (*n* = 23). This suggests that these LN patients had an immune profile similar to that of the controls (Figure 3B). The remaining 2 groups, G1 and G2, each contained 46 LN patients at baseline.

We further examined which cell subsets were driving these groups of patients with LN. The extremes of principal component (PC) 1 were defined by cells correlated with IFN-I score, suggesting that IFN response represented the main axis of variation in blood immunophenotypes (Figure 3C). IFN-I–correlated subsets, including proliferating NK cells and transitional B cells, were significantly enriched in G1 compared with the other groups (Supplemental Figure 7, C and D). Comparing IFN-I score between the 3 groups of LN, G0 displayed the lowest values, similar to controls, G1 had the highest, and G2 showed intermediate levels and the most variability (Figure 3D). Proliferating T and B cell subsets (B8, B10, B11, T11, and T13) were enriched in both groups G1 and G2 compared with G0 (Figure 3E). In contrast, G2 was marked by enrichment of granzyme B^+^ cell subsets, aligning with the negative axis of PC2, which appeared to be driven by granzyme B^+^ T cells (T4, T9, T16, T17, T18, T20, and T13) (Figure 3, C and F, and Supplemental Figure 7F).

Considering the heterogeneity of patient characteristics in this real-world cohort, we confirmed cell associations with the groups after adjusting for demographic characteristics, history of previous renal biopsy, prednisone dose, and mycophenolate mofetil (MMF) use (Figure 3G). In addition to the association of G2 with granzyme B^+^ T cells, we observed an expansion of low-density neutrophils (M3) and naive CD21^lo^ B cells (B3) in this group (Figure 3G), both of which correlated with glomerular renal activity in Figure 2. CD11c^+^ B cell clusters (B5, B9) were also linked to G2, though to a lesser extent (Figure 3G). Thus, this analysis distinguished 2 groups of immunologically active patients with LN: one (G1) with a prominent IFN signature, and a second (G2) with expanded granzyme B^+^ lymphocytes.

### Baseline blood-defined LN groups have distinct renal pathology and outcome.

**Figure 4:**
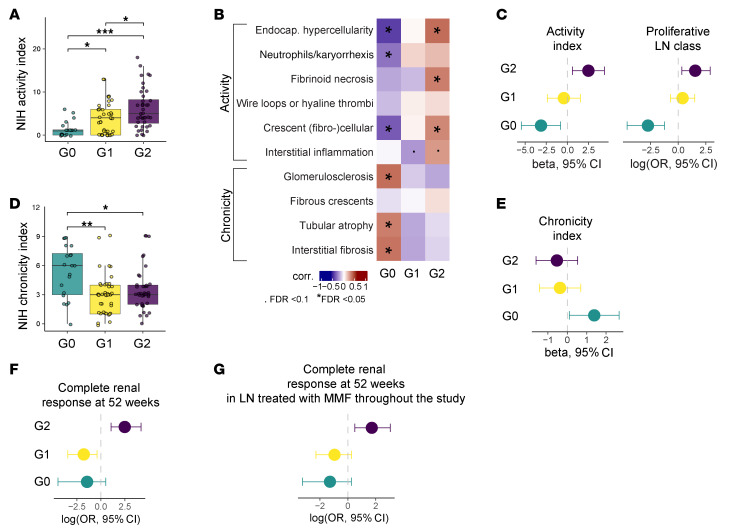
Blood-defined LN groups are associated with renal pathology and outcome. (**A**) Comparison of NIH renal activity index between blood-defined LN groups (G0: *n* = 20; G1: *n* = 40; G2: *n* = 40; LN groups were defined using *K*-means clustering based on blood immunophenotyping). Statistical significance was determined using the Kruskal-Wallis test with Dunn’s multiple comparisons. (**B**) Heatmap showing Spearman’s correlation coefficients between LN groups (one-versus-rest) and NIH renal activity/chronicity subscores (G0: *n* = 18; G1: *n* = 33; G2: *n* = 30). Adjusted significance levels are indicated (FDR < 0.05 and < 0.1). (**C**) Multivariable models evaluating one-versus-rest group associations with activity index (linear model) and proliferative class (logistic regression; reference = membranous class), adjusting for demographic factors (age, sex, ethnicity, and race), history of previous biopsy, and prednisone dose. (**D**) Comparison of NIH renal chronicity index between LN groups (G0: *n* = 20; G1: *n* = 40; G2: *n* = 40), with statistical significance determined by the Kruskal-Wallis test with Dunn’s multiple comparisons. *P < 0.05, **P < 0.01, ***P < 0.001. (**E**) Multivariable model testing one-versus-rest group associations with chronicity index (linear model), adjusting for demographic variables, history of previous biopsy, and prednisone dose. (**F**) Multivariable model testing one-versus-rest group associations with complete renal response at 52 weeks (non-complete vs. complete response), adjusting for demographic variables, history of previous biopsy, and prednisone dose. (**G**) Univariate model evaluating group associations with complete renal response at 52 weeks in patients treated with MMF throughout the study.

We next explored whether these groups of patients with LN differed in renal features. Patients in the group G0 (“control-like”) had the lowest renal activity scores, while the group G2 (“cytotoxic T–enriched”) had the highest (Figure 4A and Supplemental Table 3). Consistent with the low activity index, group G0 was more frequently classified as pure membranous, whereas G1 and G2 were more frequently classified as proliferative (85% in G2, 65% in G1, 35% in G0, *P* < 0.001). G2 status correlated significantly with endocapillary hypercellularity, active crescents, and fibrinoid necrosis, further underscoring active and more severe glomerular lesions (Figure 4B). G2 also showed a borderline positive correlation with the interstitial activity subscore (*P* = 0.01, FDR = 0.05). Compared with G1, patients in G2 at baseline had increased serum creatinine (*P* = 0.04) and UPCR (*P* = 0.009) and higher prednisone dose (*P* < 0.001). In contrast, extrarenal manifestations and serologic measures (positive anti-dsDNA and/or low complement) were not significantly different between G1 and G2 (Supplemental Table 3). Analysis of baseline SLE-associated autoantibodies, obtained from paired samples (*n* = 111) and published separately (51), was also not able to distinguish G1 from G2 (Supplemental Table 4). Adjusting for demographic variables, history of previous biopsy, and baseline prednisone dose, G2 remained significantly associated with increased renal activity indices (β coefficient [95% CI] = 2.6 [0.8–4.5], adjusted *P* value [*P*_adj_] = 0.006) and proliferative class (OR [95% CI] = 4.2 [1.4–14.1], *P*_adj_ = 0.015) compared with the other groups (Figure 4C).

Surprisingly, group G0 had the highest NIH chronicity index and subscores, whereas G1 and G2 were not significantly different (Figure 4D and Supplemental Table 3). Patients in G0 were older and more frequently had a history a previous biopsy (Supplemental Table 3), with a significant increase in past proliferative (III or IV with or without V) disease compared with G2 (65% G0, 59% G1, and 33% G2, overall *P* = 0.01, G0 vs. G2 *P* = 0.02). The majority (73%) of the patients with a membranous class in group G0 had prior history of proliferative class, a histologic conversion previously reported (52). Adjusting for demographic variables, history of previous proliferative disease, and baseline prednisone dose, G0 remained significantly associated with chronicity compared with the other groups (β coefficient [95% CI] = 1.3 [0.04–2.5], *P*_adj_ = 0.044) (Figure 4E). Together these results suggest that G0 identified a group of patients with little evidence of immune activation in blood, which is associated with minimal current renal activity, yet who may have previously had severe LN that evolved toward chronic renal damage.

We then asked whether these groups of patients had different renal outcomes. In the 88 patients with a defined renal response at week 52 (baseline UPCR ≥ 1), we observed a significant difference across groups (*P* = 0.04) and increased renal response in G2, compared with G1 (*P* = 0.03). Adjusting for demographic variables, history of previous biopsy, and baseline prednisone dose, group G2 had an increased likelihood of complete response at 1 year (OR [95% CI] = 8.5 [2.2–40.5], *P*_adj_ = 0.003) (Figure 4F). Given the variability in treatment received, we additionally tested the likelihood of response in patients treated with MMF throughout the study (MMF use at weeks 26 and 52: 52% in G0, 61% in G1, and 50% in G2), and observed a persistent association between G2 and complete response compared with others (OR [95% CI] = 5.6 [1.7–20.8], *P* = 0.007) (Figure 4G). In contrast, stratification of patients by NIH activity did not associate with renal outcome when adjusting for demographic variables (cutoff value of <3 vs. higher: OR [95% CI] = 2.35 [0.81–4.78]), supporting previous results from the AMP Network (32). These results indicate that G2, characterized by increased granzyme B^+^ T cells, neutrophils, and B cell alterations, identifies a group of patients with severe, active disease but with increased likelihood of response to standard of care, including to an MMF-based regimen.

### Baseline blood-defined LN groups differ in kidney immune cell infiltrates and urine profiles.

**Figure 5:**
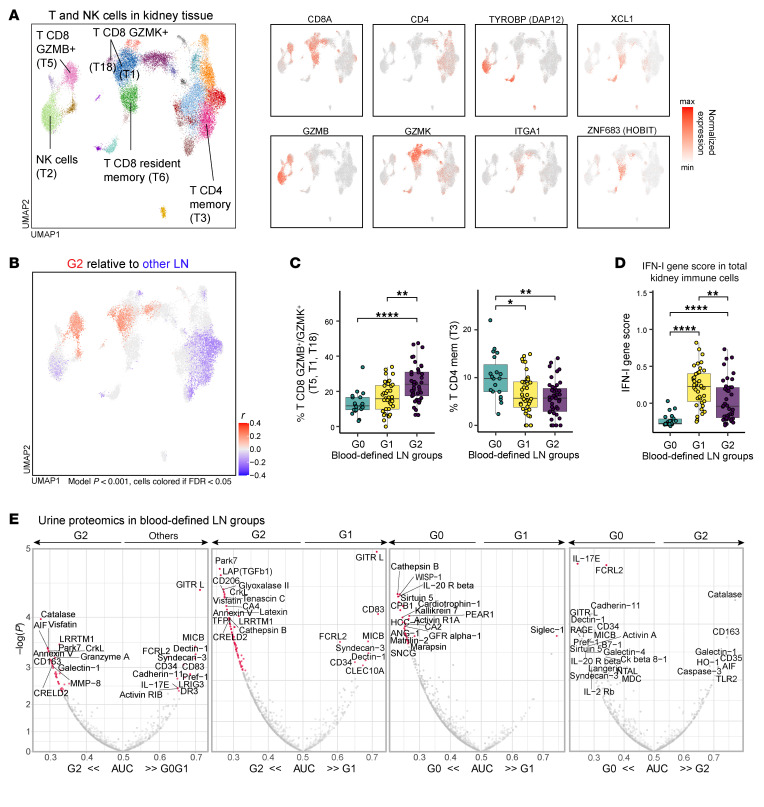
Blood-defined LN groups are associated with specific immune cell infiltration in the kidney and translate to urinary markers of inflammation. (**A**) Distribution of T cells and NK cells and selected gene expression by single-cell RNA-Seq analysis of kidney tissue from patients with LN (*n* = 101). (**B**) CNA of T-NK cells with blood-defined LN group G2 relative to others (G0 and G1) applied to paired kidney tissue samples. Cells in the UMAP are expanded (red) or depleted (blue) in patients with LN, if correlation passed FDR < 0.05. (**C** and **D**) Comparison of the proportions of CNA-identified granzyme B^+^ and a subset of granzyme K^+^ immune cell subsets, and CD4^+^ memory T cells in the kidney tissue (**C**), or IFN-I gene signature (21-gene list previously reported) in all immune cells from the kidney tissue (**D**), between blood-defined LN groups (G0: *n* = 19; G1: *n* = 40; G2: *n* = 42); *P < 0.05, **P < 0.01, ****P < 0.0001. (**E**) Comparison of each urine protein abundance between specified groups, as displayed by the performance of classification using the area under the curve (AUC). Dots colored pink passed FDR threshold of 0.10, and labels were assigned to the top associated proteins. Statistical significance was determined using Wilcoxon’s rank sum test.

Given the differences in renal activity and outcome, we hypothesized that these blood-defined LN patient groups would also differ in the composition of immune cells in the kidney. We leveraged single-cell RNA-Seq data generated on cells from kidney biopsies of 101 patients from the AMP phase II study (53, 54). Since G2 was characterized by prominent cytotoxic CD8^+^ T cell features in the blood and CD8^+^ T cells are known to accumulate in the periglomerular and interstitial space of proliferative LN kidneys (19, 55, 56), we asked whether CD8^+^ T cell infiltration in the kidney varied between these blood-defined groups. Indeed, G2 patients showed an enrichment in granzyme B^+^ CD8^+^ T cells, as well as a subset of granzyme K^+^ cells, in the kidney compared with the other LN groups (Figure 5, A–C). In contrast, G2 was not associated with CD8^+^ T cells enriched in *ITGA1*, *ZNF683*, and *XCL1*, and was depleted in *KLF2* expression (Figure 5A), features indicative of resident memory T cells (57, 58), which are also present in control kidneys (4). We also observed differences in subsets of CD4^+^ T cells in the kidney, which were mostly enriched in G0 and reduced in G2 (Figure 5, A and B). Using a 21-gene IFN-I list (12), we found that G1 patients had the highest IFN-I score in kidney immune cells, while G0 had the lowest values, mirroring the pattern seen in the blood (Figure 5D). In total, these results indicate that immunoprofiling based on blood immune cells can identify subsets of patients with LN that differ in their renal immune cell infiltrates, with specific, direct relationships between immunophenotypic features in blood and those in kidney.

To further extend the association between blood immune profiles and kidney injury, we compared the urine proteomic profiles obtained from paired samples (*n* = 115) and published separately (33). After adjusting for FDR < 0.10, we observed significant differences between groups (Figure 5E). Group G2 was enriched in granzyme A (Figure 5E), a protease expressed by granzyme K^+^ and granzyme B^+^ TCD8 cells (59), consistent with our findings from the kidney cell infiltrate. We also observed association between G2 and markers of myeloid cell degranulation (MMP-8, catalase) and activation (CD163 and galectin-1), proteins previously associated with increased NIH activity and proliferative LN class (33, 60) (Figure 5E). Compared with group G1, G2 had increased secretion of several proteins, including TGF-β1, which had been reported to be enriched in kidney tissue during proliferative LN flares and was predictive of complete renal response (6) (Figure 5E). In addition, the protein most strongly associated with G2 in contrast to G1 was Park7, a multifunctional protein that reduces oxidative stress and was suggested to protect against renal fibrosis in other conditions (61). In contrast, G1 was enriched in GITR-L and CD83, proteins expressed by antigen-presenting cells, when compared with G2 and was significantly enriched in Siglec-1 when compared with G0 (Figure 5E). Together these results highlighted the association between blood immune alterations and kidney inflammatory process in patients with LN.

### Proliferating T-B cells decrease over time in MMF-based treatment responders.

**Figure 6:**
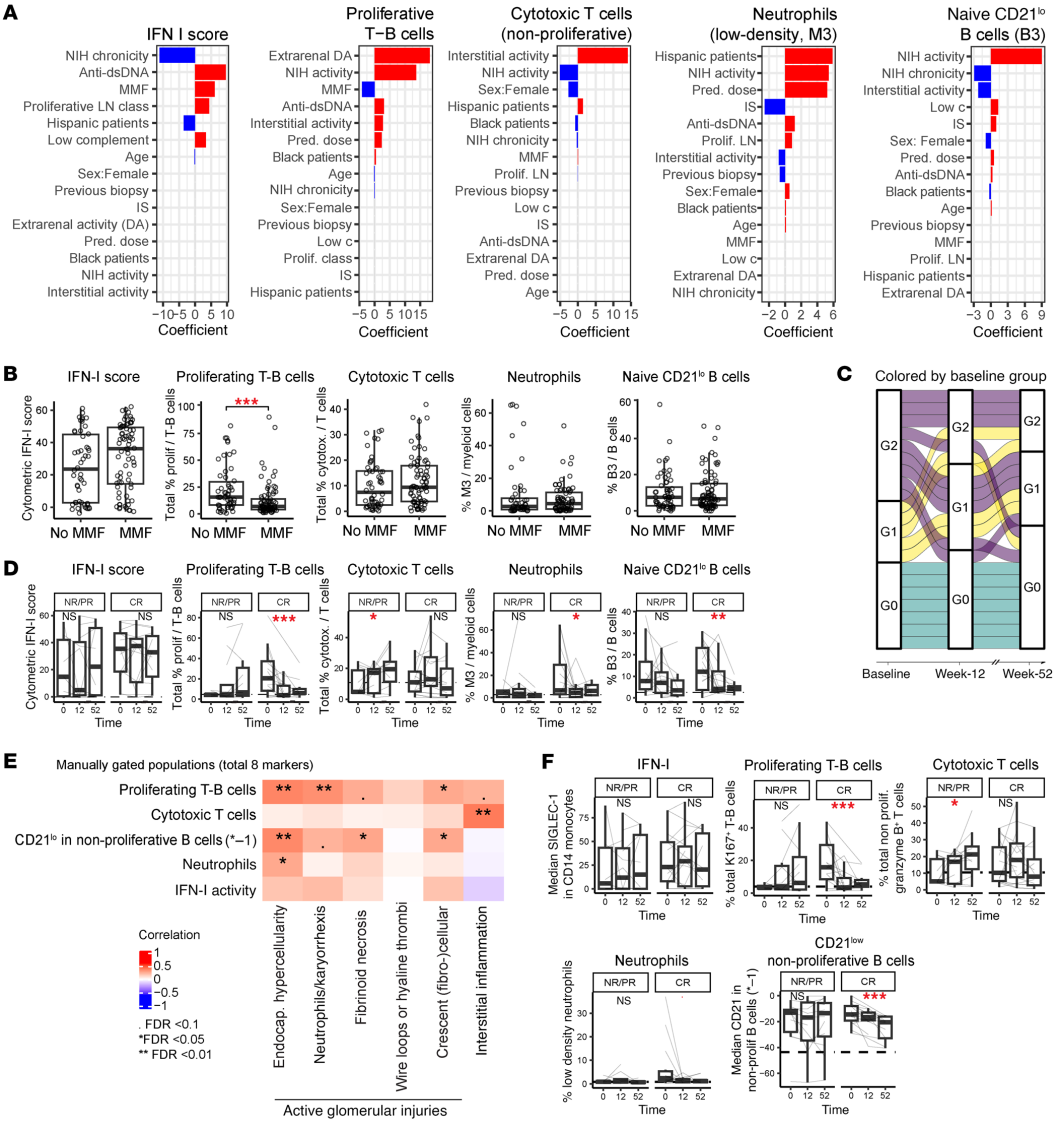
Immune cellular signature heterogeneity across and within patients with LN. (**A**) Importance and direction of the effect of demographic, clinical, and renal characteristics on immune cell signatures. The coefficients were defined by a linear model with an elastic net penalization using a 10-fold cross-validation, with the cellular signature as a response variable and the *y* axis variables as the predictor variables. IS, immunosuppressants; DA, disease activity; low c, low complement. (**B**) Comparison of immune cell signatures between treated (*n* = 80) or not treated (*n* = 59) with MMF at baseline. Statistical significance was determined using Wilcoxon’s rank sum test; ***P < 0.001. (**C**) Changes in blood-defined group membership over time. Each band represents a patient, and each patient is colored by the baseline group membership. All patients with LN with samples at 3 time points and samples stained with all 4 panels were included in this analysis (*n* = 21). (**D**) Longitudinal changes in immune cell signatures, stratified by response status (NR/PR, no response/partial response [*n* = 13]; CR, complete response [*n* = 16]) including patients with LN who were treated with MMF throughout the study. Statistical significance was determined using a mixed-effects model including patients as a random effect. *P < 0.05, **P < 0.01, ***P < 0.001. (**E**) Correlations between simplified immune cell signatures and NIH activity subscores at baseline. Statistical significance was determined using Spearman’s ρ correlation followed by FDR correction for multiple testing. For visualization purposes we showed the inversed value of median CD21 in non-proliferative B cells (written as *–1). (**F**) Longitudinal changes in simplified immune cell signatures in patients treated with an MMF-based therapy. For visualization purposes we showed the inversed value of median CD21 in non-proliferative B cells (written as *–1). Statistical significance was determined using a mixed-effects model including patients as a random effect. ^•^*P* = 0.05, *P < 0.05, **P < 0.01, ***P < 0.001.

Given our results indicating that specific blood immune cell profiles were associated with renal activity and outcome, we examined how treatment independently accounted for the variation of key blood immune signatures identified in the supervised and unsupervised analyses: IFN-I, proliferative T-B cells, non-proliferative granzyme B^+^ T cells, CD21^lo^ naive B cells, and low-density neutrophils (Figure 6A). We first confirmed that NIH activity was independently associated with proliferative T-B cells, CD21^lo^ naive B cells, and low-density neutrophils, while granzyme B^+^ T cells correlated with interstitial activity (Figure 6A). Prednisone dose (excluding high intravenous dose of 500–1,000 mg) accounted for an increase in low-density neutrophils, but did not account for the association between these cells and NIH activity index (Figure 6A). Separately, MMF was associated with decreased proliferative T-B cells (Figure 6, A and B).

We then examined the group changes longitudinally in these immune profiles. Immunologically inactive patients (group G0) at baseline uniformly remained stable over time (Figure 6C). In contrast, the majority of patients who started in G2 changed their group over time, including some transitioning to G0, suggesting that the cellular features of G2 are dynamic and can change with clinical course and treatment (Figure 6C and Supplemental Table 5). Detailed analysis of immune signatures showed that the proportion of CD21^lo^ naive B cells decreased over time in complete responders but not in partial and non-responders (Supplemental Figure 8), while immune features of IFN-I score, proliferating T-B cells, and cytotoxic T cells did not change consistently in either responders or non-responders (Supplemental Figure 8). However, the decreased trend observed in proliferative T-B cells was significant in responders when only patients with an MMF-based regimen were included, suggesting a drug effect (Figure 6D).

Finally, we confirmed that these 5 main cellular features could be captured by a simplified manual gating strategy using a limited number of canonical markers (Supplemental Figure 9). To minimize the number of markers, we measured the expression of CD21 in non-proliferative B cells, and for visualization purposes (Figure 6, E and F) we showed the inversed value, for consistency with previous graphs. Overall, this manual gating strategy, applied to the total dataset of live cells (25 × 10^6^ to 30 × 10^6^), reproduced the key patterns of cellular features associated with the LN groups (Supplemental Figure 9B), with renal disease activity (Figure 6E) and longitudinal changes, including the decrease in proliferative T-B cells and the increase in CD21 expression in non-proliferative B cells over time, supporting that these populations can readily be identified (Figure 6F).

## Discussion

Here, we have used blood cell immunoprofiling to identify immunologically distinct groups of patients with LN that differ in renal inflammation, histologic class, and outcome in a large cohort of patients who underwent a clinically indicated renal biopsy. Unsupervised modeling approaches visualized co-abundant cell populations and stratified patients with LN into 3 groups: an immunologically inactive group (G0), a predominant IFN-I group (G1), and a predominant cytotoxic T cell group (G2). Notably, conventional antibody profiling was unable to differentiate between G1 and G2, indicating that serologic approaches may miss important immunologic distinctions among patients with LN. Both immunologically active groups (G1 and G2) show increased proliferating T cells and B cells, yet these groups differ with much higher IFN-I scores in G1 and increased proportions of cytotoxic T cells, low-density neutrophils, and CD21^lo^ naive B cells in G2. These results reveal specific features that distinguish the immunologic heterogeneity among patients with LN, highlighting in particular that features of an activated cellular response characterizing the group G2 can be separated from a high IFN-I response. Further, a separate group of patients with biopsy-demonstrated LN, patients in G0, show little evidence of immune activation, with immunologic profiles that are indistinguishable from those of control patients.

LN patient groups distinguished by this blood immunophenotyping approach differed in renal histologic features and renal immune cell infiltration, indicating that blood profiling can reflect relevant intrarenal features. Group G0 patients, who appeared immunologically quiet by immunophenotyping, also showed little renal activity, although they had the highest scores for chronic renal damage. The history of prior episodes of proliferative nephritis in these patients suggests that these patients may have proteinuria due to chronic renal damage but little ongoing active inflammation. Further studies will help define whether renal biopsy could be avoided in patients with quiescent blood immune profile with known LN. While blood immunologic activity could inform renal histologic activity, detailed circulating profiling may offer additional insights into renal immune cell infiltration. We showed that high IFN-I activation signaling pathway in the kidney immune cells and in urine could be robustly captured by measurement of IFN-I score in the blood, as observed in group G1. However, the particularly high expression of IFN-I, contrasting with the variability in group G2, suggests that considering the intensity of IFN-I response and combining it with other immune cell signatures might help stratify patients more effectively than the commonly used division of IFN-high versus IFN-low patients (12, 18).

In contrast to G1, group G2 showed an expansion of granzyme B^+^ T cell subsets, as well as an expansion of low-density neutrophils and CD21^lo^ naive B cells. We identified different correlations involving specific T cell phenotypes: circulating proliferating cells (including granzyme B^+^ T cells) correlated with glomerular pathology, along with CD21^lo^ naive B cells and low-density neutrophils, whereas non-proliferating granzyme B^+^ T cells correlated with interstitial renal activity. We further demonstrated that patients in group G2 had a concomitant enrichment in paired kidney samples of granzyme B^+^ and granzyme K^+^ T cell subsets, a major driver of tissue inflammation in autoimmune diseases (62) that correlates with an extrafollicular B cell response in LN kidneys (63). A pathogenic role for CD8^+^ T cells in patients with LN has been previously suggested by the enrichment in cytotoxic and proliferating CD8^+^ T cells and production of inflammatory chemokines and IFN-γ (4), a cytokine implicated in the pathogenesis of LN disease (55, 56). Our detailed profiling approach revealed an expansion of a set of co-abundant granzyme B^+^ T cell populations, including subsets of CD8^+^ T effector memory and terminally differentiated effector memory populations, proliferating CD8^+^ T cells, CD4^+^ T cells, and CD8^–^CD4^–^ T cells, associated with patients in G2. Our results provide additional granularity to previous reports describing a general expansion of effector memory CD8^+^ T cells in the blood of SLE patients associated with disease activity (64–66) and support a direct connection between effector CD8^+^ T cell frequencies in blood and kidney from LN patients.

Notably, the G2 group with a predominant cytotoxic T signature at baseline showed an increased likelihood of response to standard of care at 1 year compared with the other groups. This association could not be reproduced by stratification by NIH activity score in this work or previous work from the AMP Network (32). This is somewhat surprising because expansion of effector CD8^+^ T cells in SLE patients and infiltrating CD8^+^ T cells in LN have been associated with more severe and refractory disease (55, 56, 66). We hypothesize that the immune features in G2 reflect aspects of immune cell activation that can effectively be suppressed by current immunosuppressive therapies, such as MMF. It will be of interest in future studies to determine whether a reduction in activated immune features observed in the blood of responders is paralleled by a reduction in immune infiltrates within the kidney after treatment. The 2 other groups of patients identified were marked by either high fibrotic histologic lesions (G0) or high IFN-I responses (G1), both signatures previously associated with poorer outcome (5). In addition, we showed that the IFN-I pathway was not reduced by standard-of-care immunosuppressive therapy, supporting previous reports in general SLE (21, 40); this may potentially contribute to the low renal response rate in this group. We observed that this IFN-I–rich environment in G1 was associated with reduced expression of TCF1, a key transcription factor that regulates T cell stemness, which could be improved in SLE patients treated with IFN-I blockade. We hypothesize that the downregulation of TCF1 in high–IFN-I settings can promote differentiation of T cells toward effector states without directly contributing to the proliferative state. In addition to impairing broadly the circulating immune profile, IFN-I can impair the function of kidney parenchymal cells (67, 68). Further studies will help define whether patients with a disease characterized by high IFN-I benefit from therapies that effectively disrupt this pathway.

Our study has limitations. Although we examined the blood immunophenotype using 4 panels with 48 proteins each, other relevant signals might be present and could help further determine key cellular features associated with the immune cells enriched in the different groups of patients, including cytokine profiles enriched in the different groups. Ongoing work on the transcriptomic profile of immune and resident kidney cells, as well as blood immune cells, will provide more detailed blood-tissue comparison, including comprehensive signals that might further distinguish these groups of patients with LN. Secondly, controls were not well matched to our cohort of patients with LN. To account for demographic differences, we systematically analyzed our data in univariate and multivariate models adjusting for age, sex, ethnicity, and race. Thirdly, SLE patients without nephritis were not included; therefore, we cannot assess cytometric features that may distinguish renal from non-renal SLE. Finally, the lack of standardized therapy in this study limited our ability to evaluate the impact of specific therapies on immunophenotypes. Despite these challenges, we were able to identify immune cell changes that were either associated with or independent of the immunosuppressive therapies. Detailed assessment in a randomized trial with standardized therapy will further help to understand the immune changes associated with the heterogeneity of response to specific therapies.

In summary, our study highlights the power of blood immunophenotyping to define subsets of patients with LN with distinct patterns of immune activation that reflect ongoing renal inflammation and injury, which provides critical evidence for future personalized treatment. This work indicates a valuable role for blood cellular biomarkers in evaluating specific dimensions of immune activity of patients with LN to gain insight into the likelihood of response to standard immunosuppressive LN therapies.

## Methods

For additional details of methods, please refer to Supplemental Methods.

### Sex as a biological variable.

Samples from both females and males were involved in this research, and sex was reported and included as a covariate in multivariable models. The high proportion of female samples in our study reflects the distribution of SLE, which disproportionately affects females (1).

### Study population and sample collection.

SLE patients (≥16 years) were recruited (2016 to 2021) at 14 sites across the United States as part of the Accelerating Medicines Partnership Rheumatoid Arthritis and Systemic Lupus Erythematosus (AMP RA/SLE) phase II study (32, 33). All SLE patients had a clinical indication for renal biopsy (UPCR >0.5) and biopsy-proven LN (class III, IV, and/or V). Healthy controls were recruited from 2 sites. Demographic and clinical data were collected at each site. Renal biopsies were scored according to the ISN/RPS classification (proliferative: class III or IV with or without V; or membranous: class V) and the NIH activity and chronicity indices (14) at each site, followed by a central read by 2 independent renal pathologists for 101 biopsies. Renal response was determined at 52 weeks as complete (UPCR <0.5, normal serum creatinine ≤1.3 mg/dL or, if abnormal, <125% of baseline, and prednisone <10 mg/d), partial (>50% reduction in UPCR without meeting UPCR criterion for complete response, normal creatinine ≤1.3 mg/dL or, if abnormal, ≤125% of baseline, and prednisone dose ≤15 mg/d), or none (33). Blood samples were collected at baseline, and, in some cases, at weeks 12 and 52. PBMCs were isolated, cryopreserved, and stored at a central biorepository. A total of 275 samples were collected from 152 patients with LN and 40 controls (Supplemental Figure 1A).

### Mass cytometry (CyTOF) analysis.

Cryopreserved PBMCs were analyzed at Brigham and Women’s Hospital using a Helios cytometry by time of flight (CyTOF) mass cytometer. Samples were distributed into 23 acquisition batches balanced for LN and controls and then thawed and stained, and mass cytometry data were generated as reported (69). Each sample was then split to be stained with 4 different panels including markers dedicated to examining B, T, myeloid, and NK cells (Supplemental Tables 6–9 and Supplemental Figures 10 and 11), with a panel priority depending on the number of cells available (B > T > myeloid > NK panel). We filtered out samples that had a total cell viability less than 50%, had 0 B cells, or were identified mostly (>90%) in one cluster (Supplemental Figure 1, A and B, and Supplemental Table 2). After quality control, data were arcsinh-transformed, transposed, and further processed using the Seurat package (version 4.3.0) (70) in R (version 4.3.1). We corrected batch effects using Harmony (71) (Supplemental Figure 1, C–E) and applied a graph-based cell neighborhood and cluster analysis (Supplemental Figure 1, F and G). Covarying neighborhood analysis (CNA) was used for disease and clinical variable association testing at a single-cell level, while controlling for clinical confounders and false discovery rate (FDR) (35). Disease-related signaling pathways were analyzed via single-cell correlations with CNA principal components.

### Cytometric IFN-I score.

For each sample stained with the T, B, and myeloid panels, median protein expressions of MX1 and ISG-15 across PBMCs and Siglec-1 in myeloid cells were standardized to control means and standard deviations, then summed to generate a score.

### TCF1 protein and TCF7 gene expression.

TCF1 protein expression was compared between subsets of T cells using the limma package (72). We then examined *TCF7* gene expression in relation to IFN-I using publicly available datasets: RNA-Seq data of naive T cells stimulated with or without IFN-β (Gene Expression Omnibus [GEO] GSE195541) (49), and single-cell RNA-Seq data of T cells from patients with active lupus before and after IFN-I receptor blockade (database of Genotypes and Phenotypes [dbGAP] as study phs003582.v1.p1) (73).

### Blood-defined LN groups.

Blood cell subset proportions were stratified using hierarchical clustering and *K*-means clustering (*K* = 3) to define immune profiles. LN groups (G0, G1, G2) were examined in PCA and UMAP spaces. Differences in immune populations and clinical parameters across groups were assessed using univariate and multivariable models.

### Single-cell RNA-Seq of kidney immune cells and urine proteomics.

We leveraged previously generated data from the AMP RA/SLE phase II study (33, 54) and included data from baseline kidney tissue or urine from patients who also had mass cytometry analysis of blood samples (patients with paired samples).

### Statistics.

The statistical tests performed are reported in the figure legends and as follows. For cross-sectional univariate analysis, we used Wilcoxon’s rank sum test to compare continuous variables between 2 groups, and the Kruskal-Wallis test followed by post hoc Dunn’s test to compare continuous or ordinal variables between more than 2 groups. Spearman’s correlation was used to test for association between 2 continuous or ordinal variables; when multiple comparisons were done, we controlled the FDR by using the Benjamini-Hochberg method. Categorical variables between groups were tested using Fisher’s exact test or χ^2^ test, based on the same size of each category. To adjust for potential confounders in cross-sectional analysis, we applied multivariable models using linear regression for continuous dependent variables, and logistic regression for categorical dependent variables. For feature selection of treatment and other clinical factors associated with immune cell signature expression variability, we applied a linear model with penalization (elastic net regression) after 10 random repeats of a 10-fold cross-validation. For longitudinal analysis, we used mixed-effects models with patients as a random effect, to account for repeated measures. A *P* value less than 0.05 was considered significant.

### Study approval.

All participants provided written informed consent before study enrollment, and human study protocols were approved in accordance with the Declaration of Helsinki by the institutional review boards at each participating site (Supplemental Methods).

### Data availability.

The results published here are in whole or in part based on data obtained from the ARK Portal (http://arkportal.synapse.org). The Accelerating Medicines Partnership (AMP) RA/SLE Network data used for this publication are available at https://doi.org/10.7303/syn65922997.1 The ARK Portal hosts data generated by a network of research teams working collaboratively to deepen the understanding of arthritis and autoimmune and related diseases. It was established by the National Institute of Arthritis and Musculoskeletal and Skin Diseases and includes data from the AMP RA/SLE program. The specific data used in this publication are available as a controlled-access dataset. Researchers seeking to use these data must submit (a) a detailed intended data use statement, and (b) a completed and signed data use certificate. These access requirements ensure responsible and ethical data sharing within the research community. Instructions for access are available at https://help.arkportal.org/help/data-use-certificate#DataUse&Acknowledgement-Acknowledgement Values underlying graphs in figures are reported in the Supporting Data Values file. All analyses can be reproduced using the publicly available versions of the R packages outlined in Methods and Supplemental Methods, and R scripts are available on request.

## Author contributions

AF, KP, MD, DW, DLK, KCK, RF, MB, PI, RC, DH, ESW, MAM, J Grossman, JLB, FPS, MI, MW, MK, CP, JAJ, MAP, and JPB recruited patients, obtained samples, and curated clinical data. DAR, JAL, FZ, AF, TME, J Guthridge, PJH, MD, DW, DLK, KCK, RF, MB, PI, RC, DH, ESW, WA, MAM, J Grossman, JLB, FPS, MI, MW, MK, CCB, JBH, DSD, CP, MBB, JHA, SR, NH, JAJ, AD, MAP, JPB, and BD contributed to the processing of samples and design of the AMP SLE study. JBH and DSD provided central histopathologic reading of the kidney biopsies. AG, JK, JP, EM, KH, BH, and JAL designed and generated mass cytometry data. AG and AH processed mass cytometry data with the support of TG and TS. AH analyzed the data with the support of JI, FZ, MGA, and DAR. TS, RB, and YC advised and supported on aspects of the data analysis. TME and NH generated the single-cell RNA-Seq data from kidney tissue, and SR and AA processed the single-cell RNA-Seq data. AF analyzed the urine proteomic data. AF, PJH, PI, MBB, JHA, SR, NH, JAJ, AD, MAP, JPB, BD, FZ, JAL, and DAR provided disease clinical and immunology input. AH and DAR wrote the initial draft. DAR, JAL, and FZ supervised the research. All authors participated in editing the final manuscript.

## Supplementary Material

Supplemental data

Supporting data values

## Version 1 06/19/2025

In-Press Preview

## Version 2 08/15/2025

Electronic publication
