# Supplementary material for: Blood immunophenotyping identifies distinct kidney histopathology and outcomes in patients with lupus nephritis
Source: J Clin Invest. 2025 Jun 19;135(16):e181034. doi: 10.1172/JCI181034 (PMC12352894; doi:10.1172/JCI181034)
Supplement: Supplemental data [file jci-135-181034-s244.pdf]

Supplemental Table 1. Baseline characteristics of all subjects included in the study and renal response to treatment at 52 weeks

	**Lupus nephritis patients**	**Controls**	***p value****[*]*
**N**	**145**	**40**	
**Age, median (IQR)**	33 (26-43)	44 (27-60)	0.02
**Female**	126 (87)	28 (70)	0.02
**Ethnicity and Race**[1]			
Hispanic or Latino	45 (31)	4/39 (10)	0.008
Black	67/128 (52)	10 (25)	0.003
**Age at first biopsy**[2]	26 (19-33)		
**Previous renal biopsy**	100 (69)		
Previous LN class I or II	12 (8)		
Previous LN class III, IV and/or V	88 (61)		
**Current renal histologic ISN class**			
Proliferative (III or IV +/- V)	102 (70)		
Membranous (V)	43 (30)		
**Activity index**[32]**, median (IQR)**	4 (1-7)		
**Chronicity index**[32]**, median (IQR)**	3 (2-5)		
**Serum creatinine**[43] **mg/ml, median (IQR)**	0.9 (0.7-1.2)		
**UPCR, median (IQR)**	2.0 (1.2-3.8)		
**Presence of cSLEDAI extrarenal**[54]	65/130 (50)		
**Positive anti-dsDNA**	96/142 (68)		
**Low C3 and/or low C4**	97/143 (68)		
**Medication at baseline**			
Hydroxychloroquine	121/144 (84)		
Prednisone dose, med (IQR)[65]	5 (0-25)		
Any immunosuppressants[76]	104/144 (75)		
Mycophenolate	82/144 (57)		
Cyclophosphamide	2/144 (1)		
**Renal response at 52 weeks (n=112)**[87]			
CR, PR, NR	31 (28), 27 (24), 54 (48)		

UPCR = urine protein/creatinine ratio. CR, PR, NR = complete, partial and non-responder. Data is presented as N (%) unless specified otherwise. The proportions, median and IQR are calculated on the total number of subjects in the group unless specified otherwise. *Chi-square, Fisher exact or Mann-Whitney tests were used when appropriate for comparison between patients and controls. [1]Self-reported; subjects with mixed ethnicity and race were counted twice. [2]n=97. [3]NIH activity and chronicity indices available in n=124. [4]n=139. [5]Presence of any clinical extrarenal features of the SLE Disease Activity Index (SLEDAI). [6]Predisone dose or equivalence at baseline in n=133, excluding one patient with missing data and seven patients receiving intravenous methylprednisolone ('pulse') dose ranging from 500-1000mg. [7]Includes immunosuppressants and biologics (azathioprine, tacrolimus, cyclosporin, methotrexate, abatacept or belimumab). [8]Renal response was determined at week 52 if baseline UPCR was ≥ 1.

Supplemental Table 2. Number of samples analyzed with the different panels after filtering for quality control

	**Lupus nephritis patients** (Total n = 227)					**Controls** (Total n = 40)				
**Panels**	**B**	**T**	**M**	**NK**	**4 panels**	**B**	**T**	**M**	**NK**	**4 panels**
**Samples**	224	213	203	191	185	40	40	40	39	39
**Subjects**	145	139	134	125	124	40	40	40	39	39
**Subjects with baseline visit**	140	131	125	116	115	40	40	40	39	39
**Subjects with follow-up visits***	49	46	45	43	42	0	0	0	0	0
at least bas. and week-12	42	38	35	35	33	0	0	0	0	0
at least week-12 and -52	34	33	32	30	27	0	0	0	0	0
at least bas. and week-52	33	31	27	26	23	0	0	0	0	0
at least bas, week-12 and -52	30	28	25	24	21	0	0	0	0	0

Samples were stained with panels designed to characterize B cells (B panel), T cells (T panel), myeloid cells (M panel) and NK cells (NK panel). All values represent the number of samples or subjects who had cells stained with each panel or with all 4 panels. If the cell counts in samples were low after thawing, the panels were prioritized as following: B panel first, T panel, M panel and NK panel last. *Include any subjects with any 2 visits (bas. = baseline).

Supplemental Table 3. Baseline characteristics and renal response at week 52 of LN patients stratified by immunophenotype subgroups (n=115 LN with samples analyzed with 4 panels)

	**LN-G0 "control-like"**	**LN-G1 "IFN-I high"**	**LN-G2 "cytotoxic T"**	***global p val. ****	***G1-G2 p val. £***
**N**	**23**	**46**	**46**		
**Age, median (IQR)**	41 (31-46)	30 (24-39)	33 (26-44)	0.007	0.06
**Female**	19 (83)	37 (80)	42 (91)	0.31	
**Ethnicity and race**					
**Hispanic or Latino**	11 (48)	7 (15)	19 (41)	0.006	0.01
**Black**	10/19 (53)	20/43 (47)	23/41 (56)	0.70	
**Age at first biopsy**	31 (24-38)	21 (18-29)	25 (20-34)	0.02	0.25
**Previous renal biopsy**	20 (87)	34 (74)	29 (63)	0.10	
**Proliferative class (III or IV +/- V)**	9 (39)	31 (67)	39 (85)	<0.001	0.09
**Activity index[1], median (IQR)**	1 (0-1)	4 (0-6)	5 (3-8)	<0.001	0.03
**Chronicity index[1], median (IQR)**	6 (3-7)	3 (1-4)	3 (2-4)	0.005	0.26
**Serum creatinine mg/ml, median (IQR)**	1.0 (0.8-1.5)	0.8 (0.7-1.0)	1.0 (0.8-1.3)	0.04	0.04
**UPCR, median (IQR)**	3.4 (1.6-4.8)	1.4 (1.0-2.7)	2.2 (1.5-4.3)	0.02	0.009
**Presence of cSLEDAI extrarenal[2]**	5 (26)	16 (37)	23 (50)	0.05	
**Positive anti-dsDNA**	5 (22)	38/45 (84)	36 (78)	<0.001	0.63
**Low C3 and/or low C4**	5 (22)	31/45 (69)	34 (74)	<0.001	0.62
**Medication**					
**Hydroxychloroquine**	19 (86)	42 (91)	34 (74)	0.08	
**Prednisone, med (IQR)[3]**	0 (0-10)	0 (0-7)	20 (5-40)	<0.001	<0.001
**Any immunosuppressants[4]**	17 (77)	39 (85)	30 (65)	0.08	
**Mycophenolate**	13 (59)	32 (70)	23 (50)	0.25	
**Renal response at week 52[5]**					
**CR, PR, NR**	3(18), 3(18), 11(65)	6(18), 9(27), 19(56)	15(41), 9(24), 13(35)	0.04	0.03

UPCR = urine protein/creatinine ratio. CR, PR, NR = complete, partial and non-responder. Data is presented as N (%) unless specified otherwise. The proportions, median and IQR are calculated on the total number of subject in the group unless specified otherwise. *Chi-square, Fisher exact or Kruskal-Wallis tests when appropriate to test for differences across the three groups. [£]Chi-square, Fisher exact or Wilcoxon rank sum test to test for differences between G1 and G2. [1]NIH activity and chronicity indices available in n=100. [2]Presence of any clinical extrarenal features of the SLE Disease Activity Index (SLEDAI). [3]Predisone dose or equivalence at baseline in n=110, excluding five patients receiving intravenous methylprednisolone ('pulse') dose ranging from 500-1000mg. [4]Includes any immunosuppressants and biologics (azathioprine, tacrolimus, cyclosporine, methotrexate, abatacept or belimumab). [5]Renal response was determined at week 52 if baseline UPCR was $\geq$ 1 (n=88).

Supplemental Table 4. Baseline antibody profiles in patients with LN stratified by immunophenotype subgroups and available autoantibody profiles previously published[1]

	**LN-G0 "control-like"**	**LN-G1 "IFN-I high"**	**LN-G2 "cytotoxic T"**	***global p val. ****	***G1-G2 p val. £***
**N**	**23**	**43**	**45**		
**Anti-dsDNA, median (IQR)**	3 (1-7)	22 (7-84)	35 (11-126)	<0.001	0.18
**Anti-chromatine, N (%) positive**	7 (30)	36 (84)	43 (96)	<0.001	0.09
**Anti-ribosomal P, N (%) positive**	1 (4)	13 (30)	15 (33)	0.017	0.82
**Anti-SSA 52kd, N (%) positive**	2 (9)	11 (26)	8 (18)	0.27	
**Anti-SSA 60kd, N (%) positive**	7 (30)	19 (44)	23 (51)	0.26	
**Anti-SSB, N (%) positive**	0	7 (16)	4 (9)	0.12	
**Anti-Sm, N (%) positive**	5 (22)	26 (60)	25 (56)	0.007	0.67
**Anti-SmRNP, N (%) positive**	11 (48)	30 (70)	28 (62)	0.21	
**Anti-RNP, N (%) positive**	6 (26)	21 (49)	28 (62)	0.018	0.28

Serum samples from the AMP phase II were screened for auto-antibodies using the BioPlex 2200 ANA kit (Bio-Rad Technologies) as previously published in : Fava, A. et al. Association of autoantibody concentrations and trajectories with lupus nephritis histological features and treatment response. Arthritis Rheumatol (2024) doi:10.1002/art.42941.
*Chi-square, Fisher exact or Kruskal-Wallis tests when appropriate to test for differences across the three groups.
£Chi-square, Fisher exact or Wilcoxon rank sum test to test for differences between G1 and G2.

Supplemental Table 5. Blood-defined group membership and treatment received at the time of blood sampling in patients with lupus nephritis who had samples at three timepoints and samples stained with all panels.

Baseline visit		Week 12		Week 52	
**group**	**treatments**	**group**	**treatments**	**group**	**treatments**
G2	HCQ,Pred60,MMF2000	G2	HCQ,Pred2.5,MMF2000	G2	HCQ,Pred60
G2	HCQ,Pred20,MMF3000	G2	HCQ,Pred15,MMF3000	G2	HCQ,Pred10,MMF3000,RTX
G2	HCQ,Pred60,MMF1000	G2	HCQ,Pred5,MMF3000	G2	HCQ,Pred2.5,MMF3000
G2	HCQ,Pred40,MMF1000	G1	HCQ,Pred10,AZA	G1	HCQ,Pred7.5,AZA
G2	HCQ,Pred60	G0	HCQ,Pred30,MMF1500	G1	HCQ,Pred8,MMF1500
G2	HCQ,Pred25	G1	HCQ,Pred5,MMF2000	G1	HCQ,Pred5,MMF2000
G2	HCQ,Pred4	G1	HCQ,Pred1250,MMF3000	G1	HCQ,MMF3000
G2	Pred5	G1	MMF3000	G0	MMF2000
G2	HCQ,Pred5	G2	HCQ,Pred40,MMF3000	G0	HCQ,MMF3000
G1	HCQ,Pred15,AZA	G2	HCQ,Pred10,CYC,RTX	G2	HCQ,Dapsone,Pred15
G1	HCQ,Pred5,MMF2500	G1	HCQ,Pred5,MMF3000,TAC	G2	Pred2.5,MMF3000,TAC
G1	HCQ,MMF1000	G1	HCQ,MMF2000	G1	HCQ,MMF3000
G1	HCQ,MMF2000	G1	HCQ,MMF2000	G1	HCQ,MMF3000
G1	HCQ,MMF1000	G2	HCQ,Pred20,CYC	G0	HCQ,Pred5,AZA
G0	HCQ,Pred20,MMF2000	G0	HCQ,Pred20,MMF2000	G0	HCQ,MMF2000
G0	HCQ, MMF3000	G0	HCQ, MMF3000	G0	HCQ, MMF3000
G0	not recorded	G0	HCQ,Pred10	G0	HCQ,Pred5,MMF2000
G0	HCQ,Pred5,AZA	G0	HCQ,Pred5,AZA	G0	HCQ,Pred2.5,AZA
G0	HCQ,MMF2000	G0	HCQ,MMF2000	G0	HCQ,MMF3000
G0	HCQ,MMF3000	G0	not recorded	G0	not recorded
G0	HCQ,Pred40	G0	HCQ,Pred5,MMF3000	G0	HCQ,Pred5,MMF2000,TAC

HCQ = hydroxychloroquine, Pred = prednisone dose or equivalent, MMF = mycophenolate mofetil, CYC = cyclophosphamide, AZA = azathioprine, TAC = tacrolimus, RTX = rituximab. Prednisone and MMF dose are speficied in the table.

Supplemental Table 6. Lineage and B-cell specific mass cytometry antibodies

Panel	Metal	Marker	Clone	Supplier	Catalog #
All	89Y	CD45	HI30	Biolegend	304045
All	111Cd	CD172ab	SE5A5	Biolegend	323802
All	112Cd	CD8a	RPA T8	Biolegend	301053
All	113Cd	CD20	2H7	Biolegend	302343
All	114Cd	CD4	RPA T4	Biolegend	300541
All	115In	CD3	UCHT1	Biolegend	300402
All	116Cd	CD56	NCAM16.2	BD Biosciences	559043
B	141Pr	CD27	O323	Biolegend	302802
B	142Nd	Bcl-6	IG191E/A8	Biolegend	648302
B	143Nd	SLAMF7	235614	R&D Systems	MAB1906
B	144Nd	CD24	ML5	Biolegend	311102
B	145Nd	CD19	HIB19	Biolegend	302247
B	146Nd	AICDA	EK2-5G9	R&D Systems	MAB39102
B	147Sm	CD86	IT2.2	Biolegend	305410
B	148Nd	CD1c	L161	Biolegend	331502
B	149Sm	CD22	HIB22	Biolegend	302502
B	150Nd	CD11c	Bu15	Biolegend	337221
B	151Eu	CD5	UCHT2	Biolegend	300602
B	152Sm	Bcl-2	100	Biolegend	658702
B	153Eu	IgD	IA6-2	Biolegend	348202
B	154Sm	CXCR5	J252D4	Biolegend	356902
B	155Gd	CD23	EBVCS-5	Biolegend	custom
B	156Gd	CD95	DX2	Biolegend	305602
B	157Gd	CD25	M-A251	Biolegend	356102
B	158Gd	CD39	A1	Biolegend	328202
B	159Tb	TLR9	S16013D	Biolegend	394802
B	160Gd	CD307d	413D12	Biolegend	340202
B	161Dy	CD138	REA929	Miltenyi Biotech	Custom (200ug)
B	162Dy	Nur77	H1648	R&D Systems	PP-H1648-00
B	163Dy	CD83	HB15e	Biolegend	305302
B	164Dy	CD79b	CB3-1	eBioscience	14-0793-82
B	165Ho	CD38	HIT2	Biolegend	303535
B	166Er	CD40	5C3	Biolegend	334304
B	167Er	CD10	HI10a	Biolegend	312223
B	168Er	IgA	IS11-8E10	Miltenyi Biotech	130-122-335
B	169Tm	Pax-5	1H9	Biolegend	649702
B	170Er	PD-L1	29E.2A3	Biolegend	329702
B	171Yb	IgG	G18-145	BD Biosciences	555784
B	172Yb	ISG15	539442	R&D Systems	MAB4845
B	173Yb	CD21	Bu32	Biolegend	354902
B	174Yb	Ki67	8D5	Cell Signalling Technology	9449BF
B	175Lu	T-bet	4B10	Biolegend	644802
B	176Yb	IgM	MHM-88	Biolegend	314502
B	194Pt	Blimp1	646702	R&D Systems	MAB36081
B	195Pt	HLA-DR	L243	Biolegend	307651
B	196Pt	CD52	HI186	Biolegend	316002
B	198Pt	IgE	MHE-18	Biolegend	325502
B	209Bi	CD11b	ICRF44	Standard Biotools	3209003B

Cells highlighted in grey are common to all panels.

Supplemental Table 7. T-cell specific mass cytometry antibodies

Panel	Metal	Marker	Clone	Supplier	Catalog #
T	141Pr	CCR6	G034E3	Biolegend	353402
T	142Nd	CD45RA	REA562	Miltenyi Biotech	130-122-292
T	143Nd	MX1	D3W7I	Cell Signalling Technology	37849BF
T	144Nd	CCR4	L291H4	Biolegend	359402
T	145Nd	PU.1	phpu13	eBioscience	14-9819-82
T	146Nd	SH2D1A	1A9	Biolegend	690702
T	147Sm	CD45RO	REA611	Miltenyi Biotech	130-124-323
T	148Nd	CXCR3	REA232	Miltenyi Biotech	130-108-022
T	149Sm	GZMK	GM26E7	Biolegend	370502
T	150Nd	TACTILE	628211	R&D Systems	MAB6199
T	151Eu	PD-1	EH12.2H7	Biolegend	329912
T	152Sm	CTLA-4	L3D10	Biolegend	349931
T	153Eu	CD69	FN50	Biolegend	310939
T	154Sm	CXCR5	J252D4	Biolegend	356902
T	155Gd	CD15s	FH6	Biolegend	368102
T	156Gd	CD8b	SIDI8BEE	eBioscience	14-5273-82
T	157Gd	CD25	M-A251	Biolegend	356102
T	158Gd	CD39	A1	Biolegend	328202
T	159Tb	TCF1	7F11A10	Biolegend	655202
T	160Gd	ICOS	C398.4A	Biolegend	313502
T	161Dy	AHR	FF3399	eBioscience	14-9854-82
T	162Dy	Nur77	H1648	R&D Systems	PP-H1648-00
T	163Dy	CCR2	K036C2	Biolegend	357202
T	164Dy	CD161	HP-3G10	Biolegend	339919
T	165Ho	FoxP3	REA1253	Miltenyi Biotech	Custom (300ug)
T	166Er	CD40L	24-31	Biolegend	310812
T	167Er	GZMB	GB11	Biolegend	custom
T	168Er	Helios	REA829	Miltenyi Biotech	130-124-521
T	169Tm	CX3CR1	REA385	Miltenyi Biotech	130-122-286
T	170Er	RORyt	REA278	Miltenyi Biotech	130-108-059
T	171Yb	CD127	eBioRDR5	eBioscience	14-1278-82
T	172Yb	GATA3	REA174	Miltenyi Biotech	130-108-061
T	173Yb	TIGIT	MBSA43	eBioscience	16-9500
T	174Yb	Ki67	8D5	Cell Signalling Technology	9449BF
T	175Lu	T-bet	4B10	Biolegend	644802
T	176Yb	CCR7	REA546	Miltenyi Biotech	130-122-300
T	194Pt	CD57	REA769	Miltenyi Biotech	130-124-525
T	195Pt	HLA-DR	L243	Biolegend	307651
T	196Pt	CD103	Ber-ACT8	Biolegend	350202
T	198Pt	CD38	HIT2	Biolegend	303535
T	209Bi	CD11b	ICRF44	Standard Biotools	3209003B

Cells highlighted in grey are common to all panels.

Supplemental Table 8. Myeloid-cell specific mass cytometry antibodies

Panel	Metal	Marker	Clone	Supplier	Catalog #
Myeloid	141Pr	Siglec-6	767329	R&D Systems	MAB2859
Myeloid	142Nd	TLR4	610015	R&D Systems	MAB6248
Myeloid	143Nd	CD36	5-271	Biolegend	336202
Myeloid	144Nd	CD64	10.1	Biolegend	305016
Myeloid	145Nd	CD163	REA812	Miltenyi Biotech	130-122-293
Myeloid	146Nd	CD74	LN2	Biolegend	326802
Myeloid	147Sm	CD86	IT2.2	Biolegend	305410
Myeloid	148Nd	CD1c	L161	Biolegend	331502
Myeloid	149Sm	CD1d	51.1	Biolegend	350321
Myeloid	150Nd	CD11c	Bu15	Biolegend	337221
Myeloid	151Eu	CD123	6H6	Biolegend	306027
Myeloid	152Sm	CD14	M5E2	Biolegend	301843
Myeloid	153Eu	CD85d	42D1	Biolegend	338713
Myeloid	154Sm	CD15	MC-480	Biolegend	125602
Myeloid	155Gd	Siglec-1	7-239	Biolegend	346002
Myeloid	156Gd	XCR1	1097A	R&D Systems	MAB8571
Myeloid	157Gd	CD16	3G8	Biolegend	302051
Myeloid	158Gd	CD39	A1	Biolegend	328202
Myeloid	159Tb	TLR9	S16013D	Biolegend	394802
Myeloid	160Gd	FPR1	350418	R&D Systems	MAB3744
Myeloid	161Dy	CD303	REA693	Miltenyi Biotech	130-124-317
Myeloid	162Dy	MARCO	Polyclonal	R&D Systems	AF7586
Myeloid	163Dy	CCR2	K036C2	Biolegend	357202
Myeloid	164Dy	CD141	M80	Biolegend	344102
Myeloid	165Ho	CD38	HIT2	Biolegend	303535
Myeloid	166Er	CLEC9A	REA976	Miltenyi Biotech	130-122-306
Myeloid	167Er	CD84	CD84.1.21	Biolegend	326002
Myeloid	168Er	HO-1	HO-1-1	ThermoFisher	MA1-112
Myeloid	169Tm	CX3CR1	REA385	Miltenyi Biotech	130-122-286
Myeloid	170Er	PD-L1	29E.2A3	Biolegend	329702
Myeloid	171Yb	CD206	19.2	BD Biosciences	555953
Myeloid	172Yb	IRF8	REA516	Miltenyi Biotech	custom
Myeloid	173Yb	CD170	1A5	Biolegend	352002
Myeloid	174Yb	Ki67	8D5	Cell Signalling Technology	9449BF
Myeloid	175Lu	CD85i	586326	R&D Systems	MAB30851
Myeloid	176Yb	CD68	Y1/82A	Biolegend	333802
Myeloid	194Pt	CD180	MHR73-11	Biolegend	312906
Myeloid	195Pt	HLA-DR	L243	Biolegend	307651
Myeloid	196Pt	FOLR2	94b/FOLR2	Biolegend	391702
Myeloid	198Pt	CD115	61708	R&D Systems	MAB329
Myeloid	209Bi	CD11b	ICRF44	Standard Biotools	3209003B

Cells highlighted in grey are common to all panels.

Supplemental Table 9. NK-cell specific mass cytometry antibodies

Panel	Metal	Marker	Clone	Supplier	Catalog #
NK	141Pr	GNLY	Polyclonal	R&D Systems	AF3138
NK	142Nd	KIR2DS1	1127B	R&D Systems	MAB8887
NK	143Nd	CD2	TS1/8	Biolegend	309202
NK	144Nd	DAP12	406288	R&D Systems	MAB5240
NK	145Nd	NKG2C	REA205	Miltenyi Biotech	130-122-278
NK	146Nd	SH2D1A	1A9	Biolegend	690702
NK	147Sm	CD7	6B7	Biolegend	343102
NK	148Nd	GZMA	CB9	Biolegend	507202
NK	149Sm	GZMK	GM26E7	Biolegend	370502
NK	150Nd	2B4	C1.7	Biolegend	329502
NK	151Eu	TCRVd1	REA173	Miltenyi Biotech	120-014-229
NK	152Sm	PSGL-1	CHO131	R&D Systems	MAB996
NK	153Eu	CD69	FN50	Biolegend	310939
NK	154Sm	TCRgd	REA591	Miltenyi Biotech	130-122-291
NK	155Gd	EOMES	WD1928	eBioscience	14-4877-82
NK	156Gd	CD8b	SIDI8BEE	eBioscience	14-5273-82
NK	157Gd	CD16	3G8	Biolegend	302051
NK	158Gd	CD39	A1	Biolegend	328202
NK	159Tb	PLZF	R17-809	BD Biosciences	Custom
NK	160Gd	NKp30	P30-15	Biolegend	325202
NK	161Dy	MR-1-tet	5-OP-RU	Biolegend	Custom
NK	162Dy	NKp80	5D12	Biolegend	346703
NK	163Dy	4-1BB	REA765	Miltenyi Biotech	130-124-527
NK	164Dy	CXCR6	K041E5	Biolegend	356002
NK	165Ho	KIR2DS2	Polyclonal	ThermoFisher	PA5-31465
NK	166Er	CD107a	H4A3	Biolegend	328602
NK	167Er	GZMB	GB11	Biolegend	custom
NK	168Er	NKp46	REA808	Miltenyi Biotech	130-124-522
NK	169Tm	CD3z	6B10.2	Biolegend	644102
NK	170Er	iNKT	6B11	Miltenyi Biotech	130-094-865
NK	171Yb	TCRVd2	REA771	Miltenyi Biotech	130-095-212
NK	172Yb	NKG2D	149810	R&D Systems	MAB139
NK	173Yb	TCRab	T10B9.1A-31	BD Biosciences	555546
NK	174Yb	Ki67	8D5	Cell Signalling Technology	9449BF
NK	175Lu	Tbet	4B10	Biolegend	644802
NK	176Yb	Perforin	dG9	Biolegend	308102
NK	194Pt	CD57	REA769	Miltenyi Biotech	130-124-525
NK	195Pt	HLA-DR	L243	Biolegend	307651
NK	196Pt	SLAMF6	292811	R&D Systems	MAB19081
NK	198Pt	CD38	HIT2	Biolegend	303535
NK	209Bi	CD11b	ICRF44	Standard Biotools	3209003B

Cells highlighted in grey are common to all panels.

AMP RA/SLE Network

Operation/Scientific

Operations – William Apruzzese, Jennifer Goff, Patrick J. Dunn
SBG – Soumya Raychaudhuri, Fan Zhang, Ilya Korsunsky, Aparna Nathan, Joseph Mears, Kazuyoshi Ishigaki, Qian Xiao, Nghia Millard, Kathryn Weinand, Saori Sakaue
LC/STAMP – PJ Utz, Rong Mao, Bill Robinson, Holden Maecker
OMRF – Judith James, Joel Guthridge, Wade DeJager, Susan Macwana

SLE

Rochester – Jennifer Anolik, Jennifer Barnas
BWH – Michael B. Brenner, James Lederer, Deepak A. Rao
Northshore – Betty Diamond, Anne Davidson, Arnon Arazi
UCSF – David Wofsy, Maria Dall'Era, Raymond Hsu
NYU – Jill Buyon, Michael Belmont, Peter Izmirly, Robert Clancy, Phillip Carlucci, Kristina Deonaraine
Einstein – Chaim Putterman, Beatrice Goilav
Hopkins – Michelle Petri, Andrea Fava, Jessica Li
MUSC – Diane L. Kamen
Cincinnati – David Hildeman, Steve Woodle
Broad – Nir Hacohen, Paul Hoover, Thomas Eisenhaure, Michael Peters, Arnon Arazi, Tony Jones, David Lieb
Rockefeller – Thomas Tuschl, Hemant Suryawanshi, Pavel Morozov, Manjunath Kustagi
UCLA – Maureen McMahon, Jennifer Grossman
UCSD – Ken Kalunian
Michigan – Matthias Kretzler, Celine Berthier, Jeffery Hodgin, Raji Menon
Texas Tech – Fernanda Payan-Schober, Sean Connery
Cedars – Mariko Ishimori, Michael Weisman

Study population

SLE patients 16 years or older were recruited between January 2016 and May 2021 at 14 clinical sites across the United States as part of the Accelerating Medicines Partnership (AMP) RA/SLE Phase II study (2, 3) if 1) they fulfilled American College of Rheumatology or Systemic Lupus International Collaborating Clinics classification criteria for SLE, 2) had a clinically-indicated renal biopsy for a UPCR > 0.5 (4, 5) and 3) had biopsy-confirmed lupus nephritis of class III, IV and/or V, based on the International Society of Nephrology/Renal Pathology Society (ISN/RPS) (6). Patients with a history of renal transplantation, a medical condition considered at risk of participating by the investigator, who were pregnant at the time of study entry, or who were exposed to rituximab within the past six months were excluded. Healthy controls were recruited from 2 of the clinical sites.

Data and sample collection

Data was collected at each site, including patient demographics (age, sex, ethnicity and race), clinical characteristics, medications, and laboratory tests. Renal biopsies were scored according to the ISN/RPS classification (proliferative : class III, IV +/- V, or membranous : class V), and the NIH activity and chronicity indices (6) at each site, followed by a central read by two independent renal pathologists for 101 biopsies. Central reads, including a detailed subscoring of the NIH activity and chronicity indices, were used if available; otherwise, total scores from site reads were used. Renal response to treatment was determined clinically at week 52 in patients with a baseline UPCR ratio ≥ 1, as : complete (UPCR <0.5, normal serum creatinine ≤ 1.3 mg/dL or, if abnormal, <125% of baseline, and prednisone ≤10mg/day), partial (>50% reduction in UPCR without meeting UPCR criterion for CR, normal creatinine ≤ 1.3 mg/dL or, if abnormal, ≤ 125% of baseline, and prednisone dose ≤ 15 mg/day), or none (3). Blood samples were collected from patients with LN and controls at baseline (week 0), and a subset of patients with LN were recollected at weeks 12 and 52. PBMCs were isolated from blood samples and cryopreserved at each site as

previously described (7). Cryopreserved PBMC were shipped to the central AMP RA/SLE Biorepository, Oklahoma Medical Research Foundation Biorepository, for storage until sample collection was complete. A total of 275 samples were collected from 152 patients with LN and 40 controls (**Supplemental Figure 1A**).

Sample staining and data acquisition by mass cytometry

PBMC samples were cryopreserved with an optimized protocol (7) and were sent to the Brigham and Women's Hospital CyTOF Antibody Resource and Core for mass cytometry (**Supplemental Figure 1A**). Samples were then randomly assigned to 23 staining and acquisition days (batches), with 20 samples processed per day, ensuring a balanced distribution of LN and control samples across all batches (**Supplemental Figure 1A**). Samples were thawed, stained and mass cytometry data was generated according to the AMP RA/SLE mass cytometry processing workflow, and as previously reported (8). Samples were thawed in a 37 °C water bath for 3 minutes and then mixed with 37 °C thawing media containing: RPMI Medium 1640 (Life Technologies #11875-085) supplemented with 5% heat-inactivated fetal bovine serum (Life Technologies #16000044), 1 mM GlutaMAX (Life Technologies #35050079), antibiotic-antimycotic (Life Technologies #15240062), 2 mM MEM non-essential amino acids (Life Technologies #11140050), 10 mM HEPES (Life Technologies #15630080), 2.5 x 10^{-5} M 2-mercaptoethanol (Sigma-Aldrich #M3148), 20 units/mL sodium heparin (Sigma-Aldrich #H3393), and 25 units/mL benzonase nuclease (Sigma-Aldrich #E1014). 100 µL aliquots of each sample post-thaw were mixed with PBS (Life Technologies #10010023) at a 1:1 ratio to be counted by flow cytometry.

Each sample was then split into 4 to be stained with 4 different panels including lineage and specific markers dedicated to examining B, T, myeloid, and NK cells (**Supplemental Tables 6-9,**

Supplemental Figure 10-11). Overall, between 0.5 – 1.0 x 10^6 cells were stained per panel for each sample. If the sample before splitting included <2x10^6, a panel priority was used: B panel > T panel > Myeloid panel > NK panel (**Supplemental Figure 1A**). All samples were transferred to a polypropylene plate (Corning #3365) to be stained at room temperature for the rest of the experiment. The samples were spun down and aspirated. Rhodium viability staining reagent (Standard BioTools #201103B) was diluted at 1:1000 and added for five minutes. 16% stock paraformaldehyde (Fisher Scientific #O4042-500) was diluted to 0.4% in PBS and added to the samples for five minutes. After centrifugation and aspiration, Human TruStain FcX Fc receptor blocking reagent (BioLegend #422302) was used at a 1:100 dilution in cell staining buffer (CSB) (PBS with 2.5 g bovine serum albumin [Sigma Aldrich #A3059] and 100 mg of sodium azide [Sigma Aldrich #71289]) for 10 minutes followed by incubation with conjugated surface antibodies (each marker was used at a 1:100 dilution in CSB, unless stated otherwise) for 30 minutes. The Harvard Medical Area CyTOF Antibody Resource and Core (Boston, MA) prepared and validated all antibodies. After centrifugation, samples were resuspended with culture media. 16% stock paraformaldehyde (Fisher Scientific #O4042-500) dissolved in PBS was used at a final concentration of 4% for 10 minutes to fix the samples before permeabilization with the FoxP3/Transcription Factor Staining Buffer Set (ThermoFisher Scientific #00-5523-00). The samples were incubated with SCN-EDTA coupled palladium barcoding reagents for 15 minutes, followed by incubation with Heparin (Sigma-Aldrich #H3149-100KU) diluted 1:10 in PBS. Samples were combined and filtered in a polypropylene tube fitted with a 40μm filter cap. Conjugated intracellular antibodies were added into each tube and incubated for 30 minutes. Cells were then fixed with 4% paraformaldehyde for 10 minutes. DNA was labeled for 20 minutes to identify single-cell events with an 18.75 μM iridium intercalator solution (Standard BioTools #201192B). Samples were subsequently washed and reconstituted in Cell Acquisition Solution (CAS) (Standard BioTools #201240) in the presence of EQ Four Element Calibration beads (Standard BioTools

#201078) at a final concentration of $1x10^6$ cells/mL. Samples were acquired on a Helios CyTOF Mass Cytometer (Standard BioTools).

Mass cytometry data processing and quality control

Raw FCS files were normalized to reduce signal deviation between samples during multi-day batch acquisitions, based on the bead standard normalization method established by Finck et al. (9). The normalized files were then compensated with a panel-specific spillover matrix to subtract cross-contaminating signals (10). These compensated files were then deconvoluted into individual sample files using a single cell-based debarcoding algorithm established by Zunder et al (11). Normalized and debarcoded FCS files were then uploaded to OMIQ software from Dotmatics (www.omiq.ai, www.dotmatics.com), as previously reported (8). Manual gating on Gaussian parameters and DNA was applied to remove debris, outlier events, and doublets. Singlet live cells were then identified by gating on bead-negative and Rhodium-negative cell events. All proteins in each panel were examined and included in further analysis, except for IgG and IgA in the B panel, due to an identified strong co-expression of both markers that was determined to be a technical limitation. The data was further evaluated for quality control by examining the proportion of viable cells, the presence and frequency of major cell types (B, T, myeloid, and NK) and the distribution of samples using unsupervised clustering with opt-SNE dimensionality reduction and PARC clustering (12), available in the OMIQ software from Dotmatics (www.omiq.ai, www.dotmatics.com). Samples were excluded from further analysis if they presented one or several quality issues: cell viability < 50%, 0 B cells, and samples identified mostly (>90%) in one PARC cluster (**Supplemental Figure 1A**, **Supplemental Table 2**).

Single-cell proteomic data transformation and batch correction

The data generated from each panel was analyzed using the same workflow: after quality control filtering, FCS files including live singlet cells were read in R (R version 4.3.1) using the flowCore

package (version 2.14.0) and were arcsinh-transformed with a cofactor of 5, as described (13). We leveraged the single-cell transcriptomic analysis pipeline from the Seurat package (version 4.3.0) (14) by transposing our transformed expression matrix, and implementing our matrix into a Seurat object with the corresponding metadata. To ensure equal representation of samples and to save computational time and resources, we randomly downsampled each sample to 10,000 cells (if a sample had < 10,000 cells, all cells were kept, **Supplemental Figure 1B**), as downsampling does not affect the sensitivity of subsequent analysis (8). We used principal component analysis (PCA) for dimensionality reduction (function RunPCA, set without approximation) from the Seurat package and then corrected batch effects with the function RunHarmony based on the Harmony (15) application programming interface implemented in the Seurat package, using the first 20 PCs (**Supplemental Figure 1C-D**). We confirmed the integration of the batches using the function compute_lisi from the LISI package (version 1.0) (15) (**Supplemental Figure 1E**).

Single-cell proteomic graph-based clustering and neighborhood analysis

After batch correction, we obtained a nearest-neighbor graph (function FindNeighbors) and clusters based on a Louvain-based algorithm (function FindCluster) from the Seurat package, using a resolution of 0.5 for all panels (14). Cells were then projected into two dimensions using the runUMAP function based on the 20 first harmonized PCs, with the following arguments: metric = cosine, min.dist = 0.08, spread = 1. We annotated each cluster as T, B (including clusters with a plasmablast phenotype), Myeloid (including clusters with a plasmacytoid dendritic phenotype, basophil phenotype, and neutrophil phenotype) or NK cells based on lineage markers in each panel (**Supplemental Figure 1F-H**). Clusters that were not attributed clearly to one of these cell types were labeled undetermined and were excluded from further analysis (< 0.2% of total live cells for each panel) (**Supplemental 1G**). As a second step, we extracted the cell type of interest in the dedicated panel (e.g., T cells in the T panel) and re-clustered the cells by applying again

the functions: RunPCA(), RunHarmony(), FindNeighbors() with a k=30, FindCluster() and RunUMAP(). For the Louvain-based algorithm, we optimized resolution for each cell type (0.8 for B cells, 0.7 for T cells, 0.3 for Myeloid cells, 0.3 for NK cells), based on the cluster distribution and manual check of expression of critical proteins in each cluster to gain the biological interpretations that made the most sense, with some level of over-clustering, as recommended (13). Cell-type specific clusters were labeled with a first letter corresponding to the panel they were extracted from, and a number based on the cluster size (e.g., the cluster "B0" corresponds to the largest cluster within B cells extracted from the B panel).

To visualize protein expression, we used arcsinh-transformed data and employed multiple plot types adapted from the Seurat package: 1) Dotplots to display all proteins within each cluster with dot color representing the average unscaled expression and dot size indicating the percentage of cells with non-zero values; 2) Violin plots to show the distribution of a selection of proteins, where the minimal and maximal thresholds were data-driven by the cells expressing the lowest and highest expression; 3) Feature plots to map a selection of protein expression in the UMAP space, with the same min-max threshold definition than in 2).

Cytometric type I interferon score calculation

To define a cytometric type I IFN score, we first identified any proteins in our panels that had been previously and repeatedly used as a gene or a protein associated with type I interferon signaling (16–18). Median expression of MX1 and ISG15 was calculated amongst total live cells, as the expression was broadly observed for both markers, and median SIGLEC-1 was measured amongst myeloid cells, as no other cell type expressed it (**Supplemental Figure 3C**). For each sample stained with these three panels (T, B and myeloid), the values for each marker were standardized to the mean and standard deviation of the controls and then summed to obtain a score.

TCF1 protein, *TCF7* gene expression and stemness score association with interferon

To identify the differential expression markers between two T CD4 naïve clusters associated with type I interferon signature, we used the limma package, a method used previously to evaluate differential expression in mass cytometry dataset (19), to compute the p values for each marker included in the T panel. After identifying TCF1 as the most differentially expressed marker, we examined it's expression (gene *TCF7*), as well as the expression of Ki67 (gene *MKI67*, a marker of proliferation), in naïve T $CD4^+$ and T $CD8^+$ cells after stimulation with CD3/CD28 with or without IFN beta, using a publicly available RNAseq dataset (GSE195541) (20).

In addition, we used a previously reported dataset including single-cell RNAseq data of T cells from 7 patients with active lupus erythematosus before and after treatement with an IFN receptor blockade (anifrolumab) (21). Raw data is available through dbGAP as study phs003582.v1.p1, and sample collection, data generation and processing is detailed in (21). We examined the expression of *TCF7* and *LEF1* in T cells, two genes associated with T cell stemness, before and after treatement (22). The stemness score was calculated using the AddModuleScore function from the Seurat package. Median expression of the stemness score for each sample was used to determine statistical difference before and after treatment, using a paired Wilcoxon test.

Supervised approaches for disease or renal features association testing

To identify LN-associated cells or histology features-associated cells, we applied co-varying neighborhoods analysis (CNA) (23) using its r version (rcna package, version 0.0.99) implemented in the Seurat package (association.Seurat function). CNA defines data-driven neighborhoods (here referring to small regions of cells sharing proteomic similarities) and measures the relative abundance of cells from each sample across all neighborhoods. By further applying PCA to the neighborhood abundance matrix, CNA identifies dominant axes of variation

(NAM-PCs) in cell neighborhood abundance across samples and further allows to test for clinical association including potential covariates, in a linear model. For each model run, the function provides the global CNA p-value, which is defined through a permutation test, and the cell-neighborhoods that passes a threshold of FDR<0.05 (23). Global CNA p value and significant local associations (FDR < 0.05) were reported and mapped in the UMAP space. CNA associations were validated at a cell subset (cluster) level by comparing its median cell correlation coefficient with the odds ratio defined by a separate single-cell statistical approach, the mixed-effects association of single cells (MASC) (24) (**Supplemental Figure 12A**); notably, CNA was robust to small cluster outliers (**Supplemental Figure 12B**). To identify disease-associated signaling pathway activation, we assessed the correlation between each marker's expression and the axis of greatest variance (NAM-PC1) defined by each CNA model. This was done at a single-cell level using Spearman's correlation coefficient, calculated with the rcorr function from the Hmisc package (version 5.1-1).

Unsupervised approaches to identify immune cell profiles at a sample-level

To identify hidden circulating cellular profiles at a sample-level, we applied unsupervised machine learning approaches using the proportions of each cell subsets for each PBMC sample (e.g. % B0 / total B cells, % B1 / total B cells, % T0 / total T cells, etc). To prevent signals from cell cluster outliers, we removed cell subset clusters that included more than 50% of samples with a zero value (examples shown in **Supplemental Figure 12B**); we excluded three T cell clusters (T21, T22, and T23), three B cell clusters (B14, B15, and B16) and two myeloid clusters (M10 and M12). Only samples stained with all panels (T, B, Myeloid and NK) were included in these analysis. We obtained a matrix of 55 cell subset (=cell cluster) proportions with each sample represented by a row and each cell subset proportion represented by a column.

Hierachical clustering of cell subset co-abundance matrix. For this analysis, we first obtain Spearman's rho correlation coefficient and p values between all cell subset proportions and the cytometric interferon score including baseline samples from patients with LN. Spearman's rho correlation coefficient and p values were calculated using the the rcorr function from the Hmisc package, and the FDR were obtained using the p.adjust function from the stats package (version 4.3.1). Correlation coefficients were then organized using a hierarchical unsupervised clustering algorithm with Ward distance method, and visualized using the R package corrplot (version 0.92) (**Figure 3A, Supplemental Figures 6A**).

K-means grouping of samples (= refered to as LN groups). For the second unsupervised approach, we applied a K-means clustering algorithm to group LN and control samples based on their scaled proportions of cell subsets. We tested the stability of different numbers of K (=groups) by repeating 1000 K-means clustering on a resampled dataset without replacement and confirmed > 80% of stability for our final approach, using the ConsensusClusterPlus package (version 1.66.0) (25) (**Supplemental Figure 7A**). The final K-means clustering was performed with a K=3 using the kmeans function from the stats package, resulting in the groups (= blood-defined groups) labelled as G0, G1 and G2. We examined that the differences in the groups were reflected in the PCA and UMAP spaces using the umap function from the uwot package (version 0.1.16) (**Figure 3B, Supplemental Figure 7B**). To identify the cells driving the differences between these groups, 1) we examined the cell subset loadings in the first 2 PCs (**Figure 3C**), 2) we run cell-type specific CNA comparing each group with the others including patients with LN at baseline and used a heatmap to combine the results (**Figure 3G**) and 3) we compared the proportions of a selection of cell subsets, either combined or individually (**Figure 3E-F, Supplemental Figure 7D-F**). For visualization of CNA results, we plotted the frequency of cells within each cell subset passing the FDR threshold of <0.05. All CNA models with cells passing FDR had a global p value of < 0.05.

Baseline demographic, clinical and histological data were compared between these newly defined groups of samples from patients with LN (= LN groups) using univariate and multivariable models, as indicated (**Figure 4**, **Supplemental Table 3**).

SLE-associated auto-antibodies analysis and association with K-means blood-defined groups

SLE-associated auto-antibodies dataset was previously generated and reported for patients with LN included in the AMP phase II study (26). Briefly, serum samples were screened for autoantibody speficities using the BioPlex 2200 ANA kit (Bio-Rad Technologies). Anti-dsDNA was reported in international units per milliliter and all others as an index based on fluorescence intensity; autoantibody positivity was determined per the manufacturer's recommendations, as previously described (26). To investigate whether autoantibodies were associated with blood-defined groups, we analyzed patients with both paired mass cytometry PBMC data and autoantibodies data, allowing us to assign blood-defined groups to corresponding serum samples at the patient level. To examine differences across the three groups, we used Kruskall-Wallis test for anti-dsDNA and Chi-square or Fisher exact for all other antibodies. To examine differences between G1 and G2, we used Wilcoxon rank sum test for anti-dsDNA and Chi-square or Fisher exact for all other antibodies (**Supplemental Table 4**).

Single-cell kidney immune cells analysis and association with K-means blood-defined groups

As part of the AMP phase II study, baseline human kidney biopsies were cryopreserved, thawed, dissociated, sequenced and processed as described (27). Briefly, following alignment, dimensionality reduction was achieved by identifying variable genes and running PCA, followed by batch correction using Harmony (15), graph-based clustering using Seurat (version 4.1.0)(14).

T and NK cell clusters were identified based on the expression of known lineage markers (Al Souz *et al.*, manuscript in preperation). To investigate whether kidney immune cells were associated with blood-defined groups, we analyzed patients with both paired mass cytometry PBMC data and single-cell RNA-seq kidney data, allowing us to assign blood-defined groups to corresponding kidney samples at the patient level. We then applied CNA (23) to the kidney single-cell RNA-seq data to identify kidney T cell populations associated with blood-defined groups at a single-cell level. Results were further confirmed by comparing the proportions of kidney T cell clusters of interest between blood-defined groups. To evaluate type I IFN signaling between blood-defined groups, we used the AddModuleScore function (Seurat package) to define type I IFN score based on a previously described 21-gene list (16), and compared the gene type I IFN score in the kidney immune cells between the blood-defined groups (**Supplemental Figure 5D**).

Urine proteomics analysis and association with K-means blood-defined groups

We further leveraged the urine proteomic dataset previously generated and reported for patients with LN included in the AMP phase II study (3). Briefly, the screening was performed using an extended version of the Kiloplex Quantibody (RayBiotech) and the concentration of each analyte was normalized by urine creatinine to account for urine dilution. To investigate whether urine proteomic were associated with blood-defined groups, we included patients with both paired mass cytometry PBMC data and urine proteomic data, allowing us to assign blood-defined groups to corresponding urine samples at a patient level. We compared the abundance of urine proteins in $pg_{protein}/mg_{creatinine}$ between blood-defined groups of patients with LN using a Wilcoxon rank sum test and adjusting for FDR, with a threshold of < 0.10 and < 0.25 as indicated (**Supplemental Figure 5E**).

Statistics

In addition to the statistical testing mentioned above, we used non-parametric tests for cross-sectional univariate analysis at baseline: Wilcoxon rank sum test to compare continuous or ordinal variables between two groups, Kruskal-Wallis test followed by post hoc Dunn's test to compare continuous or ordinal variables between more than two groups, or Spearman's rho to assess the correlation between two continuous or ordinal variables (using wilcox.test, kruskal.test, dunn_test, cor.test with the method spearman functions in r). To compare categorical variables between groups, we used Fisher exact test and Chi-squared test depending on the size of the tested categorizes (using fisher.test and chisq.test functions in r). For multivariable models, we used linear regression models or generalized linear models for logistic regression (lm and glm function in r) to test for association with a continuous or categorical variable, respectively, including the covariate as indicated. To compare LN groups using logistic regression models, we applied a one-vs-rest strategy to examine the specific characteristic of one group compared to the others. To test for clinical and renal factors associated with the variation of selected cell subsets of interest, we applied a linear model with penalization using an elastic net regression (glmnet function used with the caret package) after 10 random repeats of a 10-fold cross-validation, including the cell proportion as the response variable and the clinical and renal variables as 'predictor' variables. All predictor variables were normalized to a fixed range between 0 and 1. For all above cross-sectional analysis, missing clinical data or samples were excluded from statistical testing. For longitudinal data, we used mixed effect models (lmer function from the lme4 package, version 1.1-34) to test for change over time by including each patient as a random factor to account for paired samples. For longitudinal data, we included all patients with a defined response status at 1 year who had at least two separate samples. All statistical tests were two-sided. For data visualization, we used the packages ggplot, ComplexHeatmap and corrplot. All analysis were performed on R version 4.3.1. A p value less than 0.05 was considered significant.

Study approval

All participants provided written informed consent before study enrollment, and human study protocols were approved in accordance with the Declaration of Helsinki by the institutional review boards (IRBs) at each participating sites, which included: Johns Hopkins University, New York University, University of Rochester Medical Center, Oklahoma Medical Research Foundation, University of Cincinnati, Albert Einstein College of Medicine, University of California San Francisco, Northwell Health, Medical University of South Carolina, Texas University El Paso, University of Michigan, University of California San Diego, University of California Los Angeles, Cedars-Sinai Medical Center.

References

1. Hoover PJ, Costenbader KH. Insights into the epidemiology and management of lupus nephritis from the US rheumatologist's perspective. *Kidney Int*. 2016;90(3):487–492.

2. Izmirly PM, et al. Longitudinal patterns and predictors of response to standard-of-care therapy in lupus nephritis: data from the Accelerating Medicines Partnership Lupus Network. *Arthritis Res Ther*. 2024;26(1):54.

3. Fava A, et al. Urine proteomic signatures of histological class, activity, chronicity, and treatment response in lupus nephritis. *JCI Insight*. 2024;9.

4. Fanouriakis A, et al. 2019 Update of the Joint European League Against Rheumatism and European Renal Association-European Dialysis and Transplant Association (EULAR/ERA-EDTA) recommendations for the management of lupus nephritis. *Ann Rheum Dis*. 2020;79(6):713–723.

5. Kidney Disease: Improving Global Outcomes (KDIGO) Glomerular Diseases Work Group. KDIGO 2021 Clinical Practice Guideline for the Management of Glomerular Diseases. *Kidney Int*. 2021;100(4S):S1–S276.

6. Bajema IM, et al. Revision of the International Society of Nephrology/Renal Pathology Society classification for lupus nephritis: clarification of definitions, and modified National Institutes of Health activity and chronicity indices. *Kidney Int*. 2018;93(4):789–796.

7. Bocharnikov AV, et al. PD-1hiCXCR5- T peripheral helper cells promote B cell responses in lupus via MAF and IL-21. *JCI Insight*. 2019;4(20).

8. Inamo J, et al. Deep immunophenotyping reveals circulating activated lymphocytes in individuals at risk for rheumatoid arthritis. *J Clin Invest*. 2025;135(6).

9. Finck R, et al. Normalization of mass cytometry data with bead standards. *Cytometry A*. 2013;83(5):483–494.

10. Chevrier S, et al. Compensation of Signal Spillover in Suspension and Imaging Mass Cytometry. *Cell Syst*. 2018;6(5):612-620.e5.

11. Zunder ER, et al. Palladium-based mass tag cell barcoding with a doublet-filtering scheme and single-cell deconvolution algorithm. *Nat Protoc*. 2015;10(2):316–333.

12. Stassen SV, et al. PARC: ultrafast and accurate clustering of phenotypic data of millions of single cells. *Bioinformatics*. 2020;36(9):2778–2786.

13. Nowicka M, et al. CyTOF workflow: differential discovery in high-throughput high-dimensional cytometry datasets. *F1000Res*. 2017;6:748.

14. Hao Y, et al. Integrated analysis of multimodal single-cell data. *Cell*. 2021;184(13):3573-3587.e29.

15. Korsunsky I, et al. Fast, sensitive and accurate integration of single-cell data with Harmony. *Nat Methods*. 2019;16(12):1289–1296.

16. Jayne D, et al. Phase II randomised trial of type I interferon inhibitor anifrolumab in patients with active lupus nephritis. *Ann Rheum Dis*. 2022;81(4):496–506.

17. Nakano M, et al. Distinct transcriptome architectures underlying lupus establishment and exacerbation. *Cell*. 2022;185(18):3375-3389.e21.

18. Huijser E, et al. MxA is a clinically applicable biomarker for type I interferon activation in systemic lupus erythematosus and systemic sclerosis. *Rheumatology*. 2019;58(7):1302–1303.

19. Weber LM, et al. diffcyt: Differential discovery in high-dimensional cytometry via high-resolution clustering. *Commun Biol*. 2019;2:183.

20. Sumida TS, et al. Type I interferon transcriptional network regulates expression of coinhibitory receptors in human T cells. *Nat Immunol*. 2022;23(4):632–642.

21. Law C, et al. Interferon subverts an AHR-JUN axis to promote CXCL13+ T cells in lupus. *Nature*. 2024;631(8022):857–866.

22. Escobar G, Mangani D, Anderson AC. T cell factor 1: A master regulator of the T cell response in disease. *Sci Immunol*. 2020;5(53):eabb9726.

23. Reshef YA, et al. Co-varying neighborhood analysis identifies cell populations associated with phenotypes of interest from single-cell transcriptomics. *Nat Biotechnol*. 2022;40(3):355–363.

24. Fonseka CY, et al. Mixed-effects association of single cells identifies an expanded effector $CD4^+$ T cell subset in rheumatoid arthritis. *Sci Transl Med*. 2018;10(463):eaaq0305.

25. Wilkerson MD, Hayes DN. ConsensusClusterPlus: a class discovery tool with confidence assessments and item tracking. *Bioinformatics*. 2010;26(12):1572–1573.

26. Fava A, et al. Association of autoantibody concentrations and trajectories with lupus nephritis histologic features and treatment response. *Arthritis Rheumatol*. 2024;76(11):1611–1622.

27. Hoover PJ, et al. Intrarenal myeloid subsets associated with kidney injury are comparable in mice and patients with lupus nephritis [preprint]. https://doi.org/10.1101/2023.06.24.546409. Posted on bioRxiv June 25, 2023

Supplemental Figure 1

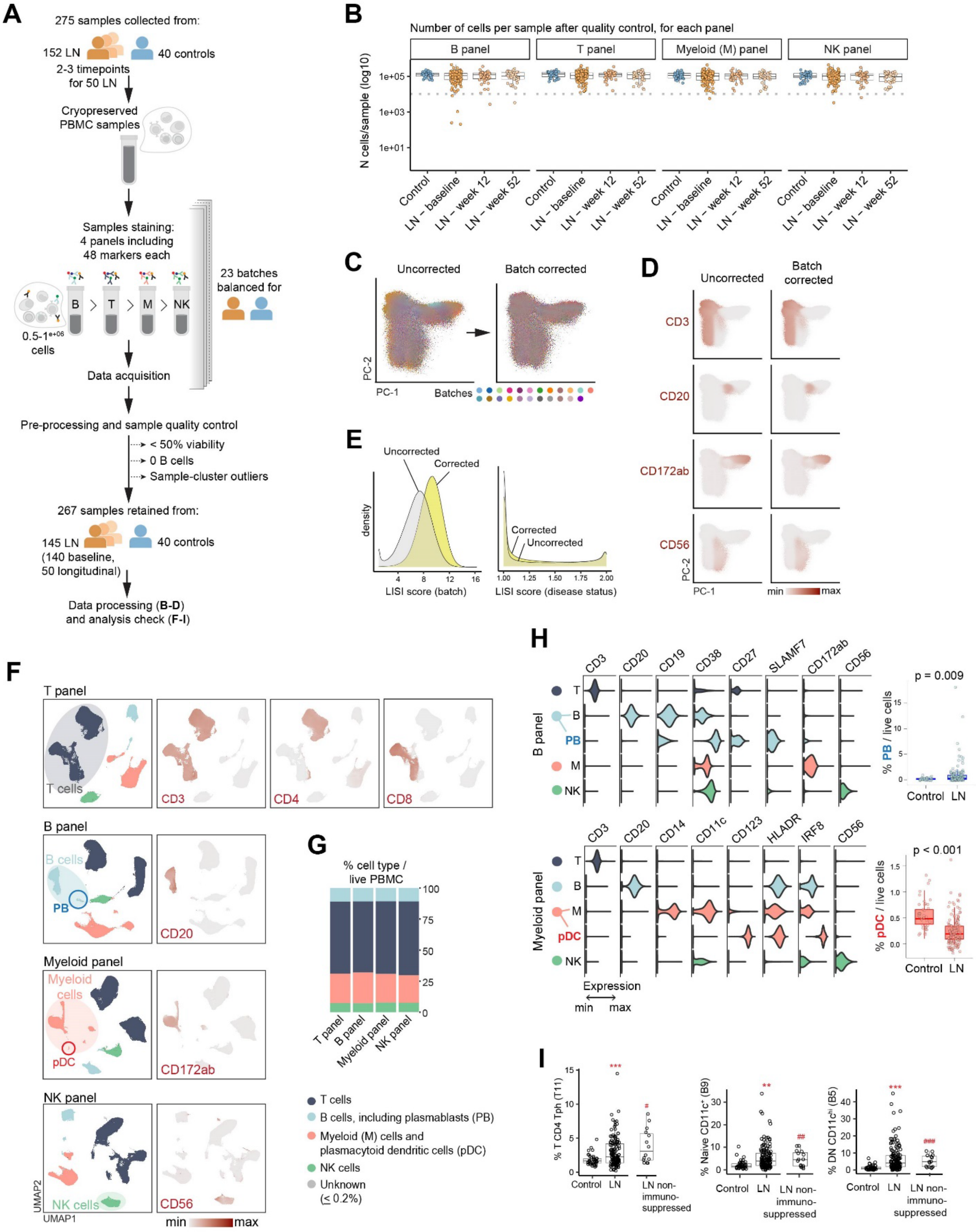

Supplemental Figure 1. Overview of blood immunophenotyping by mass cytometry in a cohort of lupus nephritis (LN). (**A**) Sample processing and quality control pipeline. (**B**) Number of cells analyzable, after filtering for quality control, in each panel and stratified by disease status and timepoints. The dotted grey line represents the threshold of 10,000, used for downsampling. (**C**) Representative example of distribution of cells stained with the T panel in the first two principal components, before and after batch correction. (**D**) Expression of key markers before and after batch correction in the T panel. (**E**) Local inverse Simpson's index (LISI) scores per cell in the T panel measuring the mixture of cells per batch or disease status (SLE versus control). Increased index represents increased mixture of cells. (**F**) Identification of major immune cell types within peripheral blood mononuclear cells from 267 samples (from 145 patients with LN and 40 controls) stained with four different panels, in the Uniform Manifold Approximation and Projection (UMAP) space. Key lineage markers by cells are displayed for the four panels. (**G**) Proportion of the main cell types, amongst total peripheral blood mononuclear cells, stratified by panel. (**H**) The left panels show key marker expression by the main cell types, and more specifically, by plasmablasts (PB) and plasmacytoid dendritic cells (pDC). The right panels shows the proportions of PB and pDC when comparing all baseline LN patients (n=140 in the B panel and n=125 in the myeloid panel) and controls (n=40); p value obtained by Wilcoxon signed-rank test. (**I**) Comparison of proportion of specific cell subsets (% Tph amongst T cells and % of $CD11c^+$ B cells amongst B cells) between 40 controls and baseline LN patients (**p<0.01, ***p<0.001 by linear regression adjusting for age, sex, ethnicity and race) or between controls and 15 LN without immunosuppression and prednisone $\leq$ 5mg ($^{\#}$p<0.05, $^{\#\#}$p<0.01, $^{\#\#\#}$p<0.001 by Wilcoxon signed-rank test).

Supplemental Figure 2

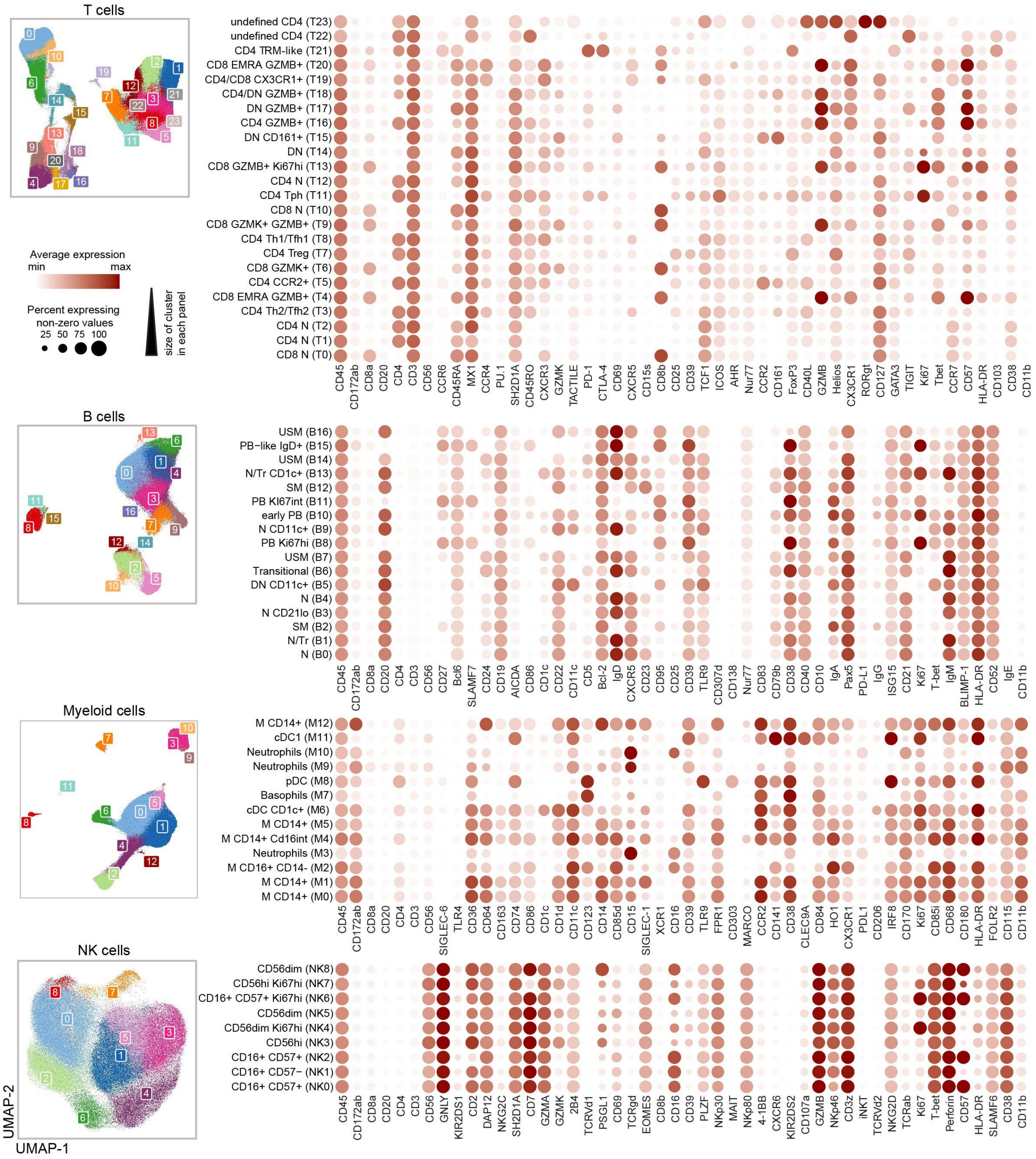

Supplemental Figure 2. Cell-type specific clustering and marker expression per cluster. Cell type specific clusters colored in the UMAP space. All proteins included in each panel are shown in the dotplots. Clusters are ordered by size (number of cells) from bottom to top, for each panel.

Supplemental Figure 3

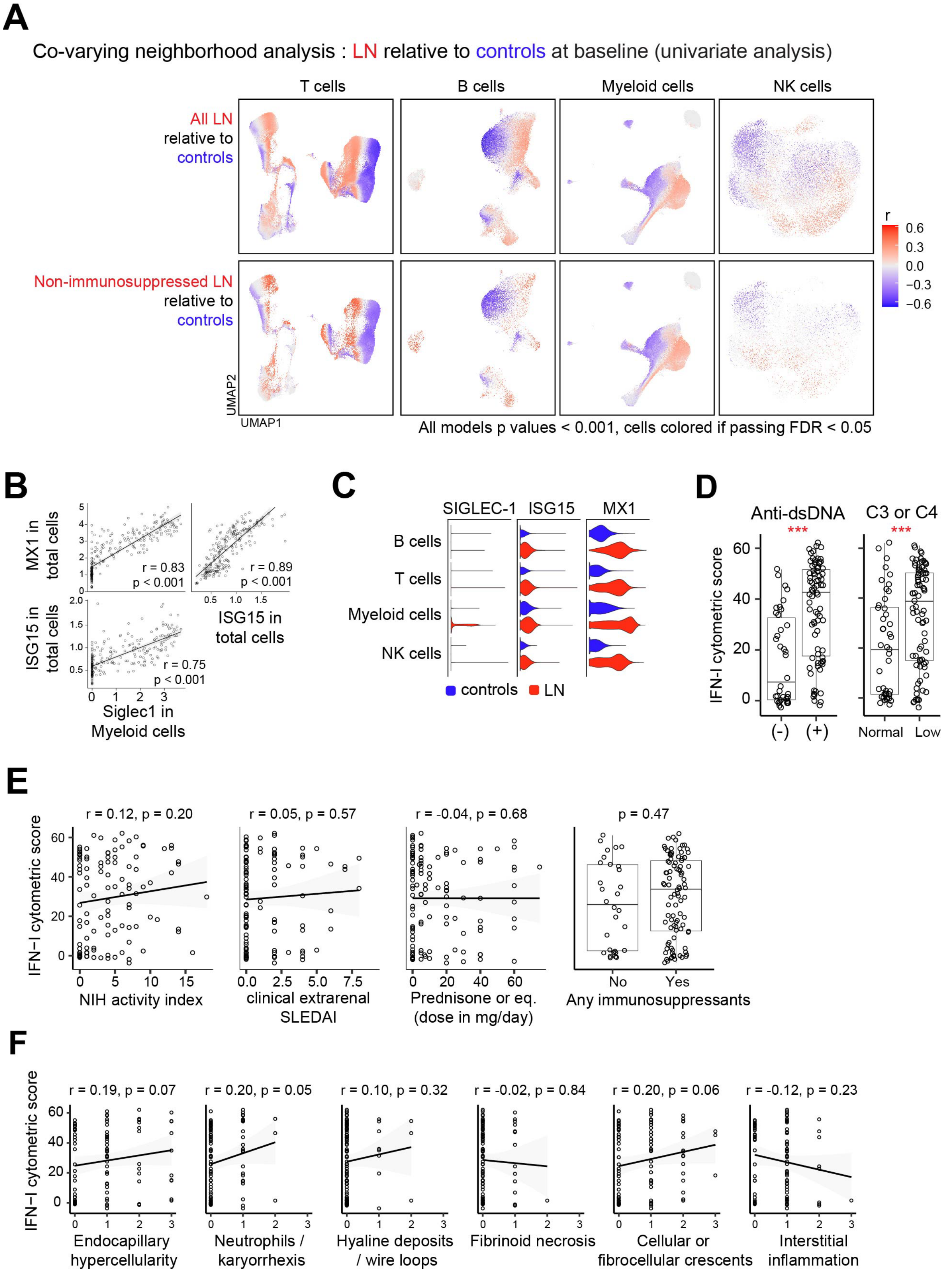

Supplemental Figure 3. Comprehensive identification of immune alterations in lupus nephritis (LN) patients reveal a proteomic type I interferon signature. (**A**) Identification of cells associated with all patients with LN (upper row) or with a subgroup of patients without immunosuppressive therapy and prednisone dose maximum 5 mg at the time of sample collection (max n=15 in the B panel and min n=13 in the NK panel), relative to controls in univariate analysis. (**B**) Correlation between the median expression per sample of MX1 and ISG amongst total live cells and SIGLEC-1 amongst myeloid cells (Spearman's rho correlation). (**C**) Distribution of the level of expression of type I interferon induced proteins in major cell types. (**D**) Comparison of a combined type I interferon score (sum of normalized MX1 and ISG15 in live cells and SIGLEC-1 in myeloid cells) in LN patients with serologic parameters. (**G**) Lack of association between cytometric type I interferon score and clinical or histologic characteristics, (**H**) including with the NIH activity subscores, using Wilcoxon rank-sum test or Spearman's rho correlation.

Supplemental Figure 4

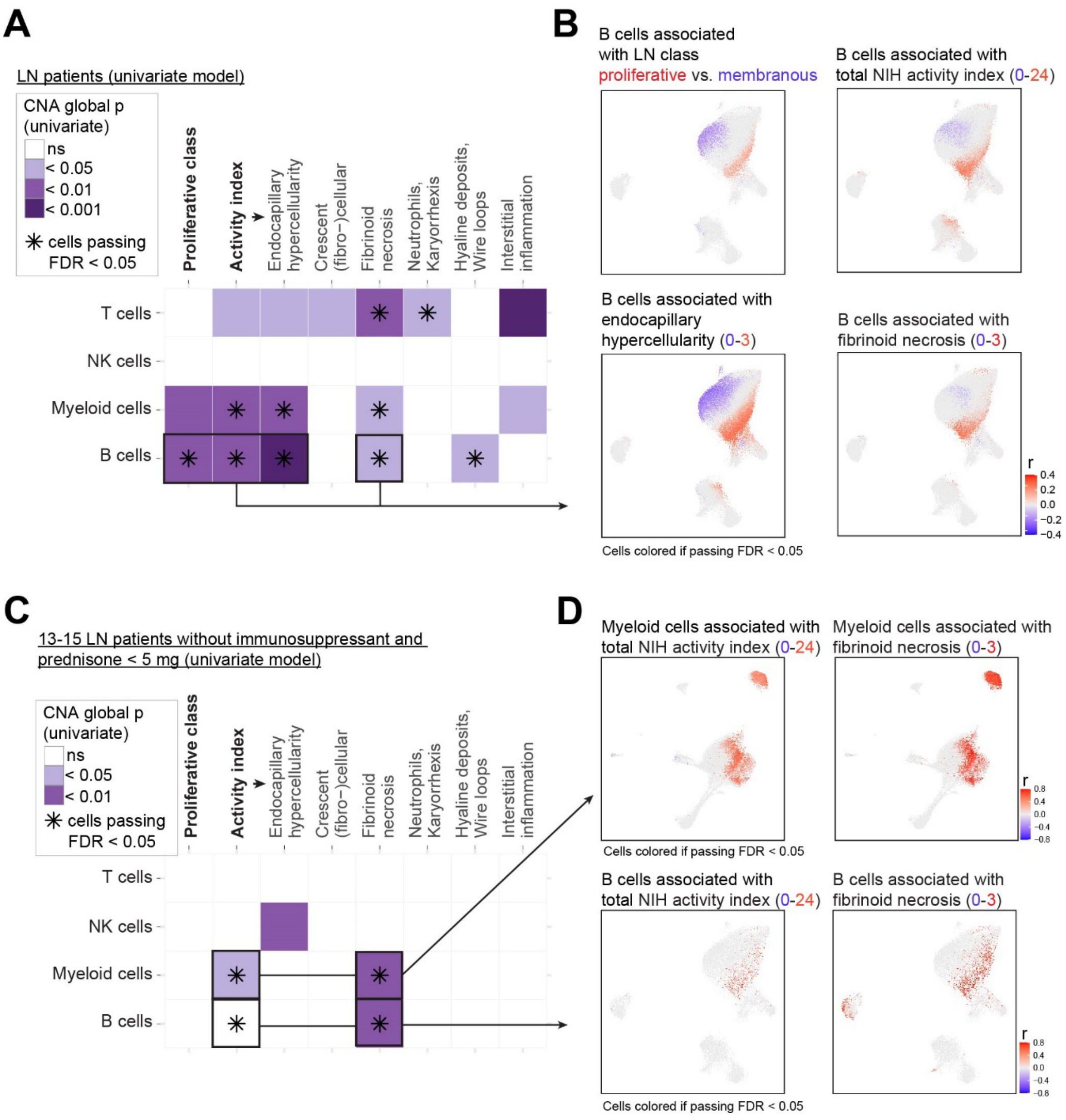

Supplemental Figure 4. Circulating cell-type specific alterations are associated with histologic patterns of active LN. (**A**) Summary of the results testing the association within each blood cell type (y axis) and different histologic patterns of LN disease (x axis). Statistical significance mentioned in the graph were determined using a univariate covarying neighborhood analysis (CNA). (**B**) Selection of detailed CNA results between B cell alterations and specified histologic characteristics. (**C**) Summary of CNA results including only LN patients with no immunosuppressive therapy and prednisone $\leq$ 5mg with (**D**), representative detailed results in the myeloid and B panels. Multiple testing is adjusted using the false-discovery rate (FDR) as indicated.

Supplemental Figure 5

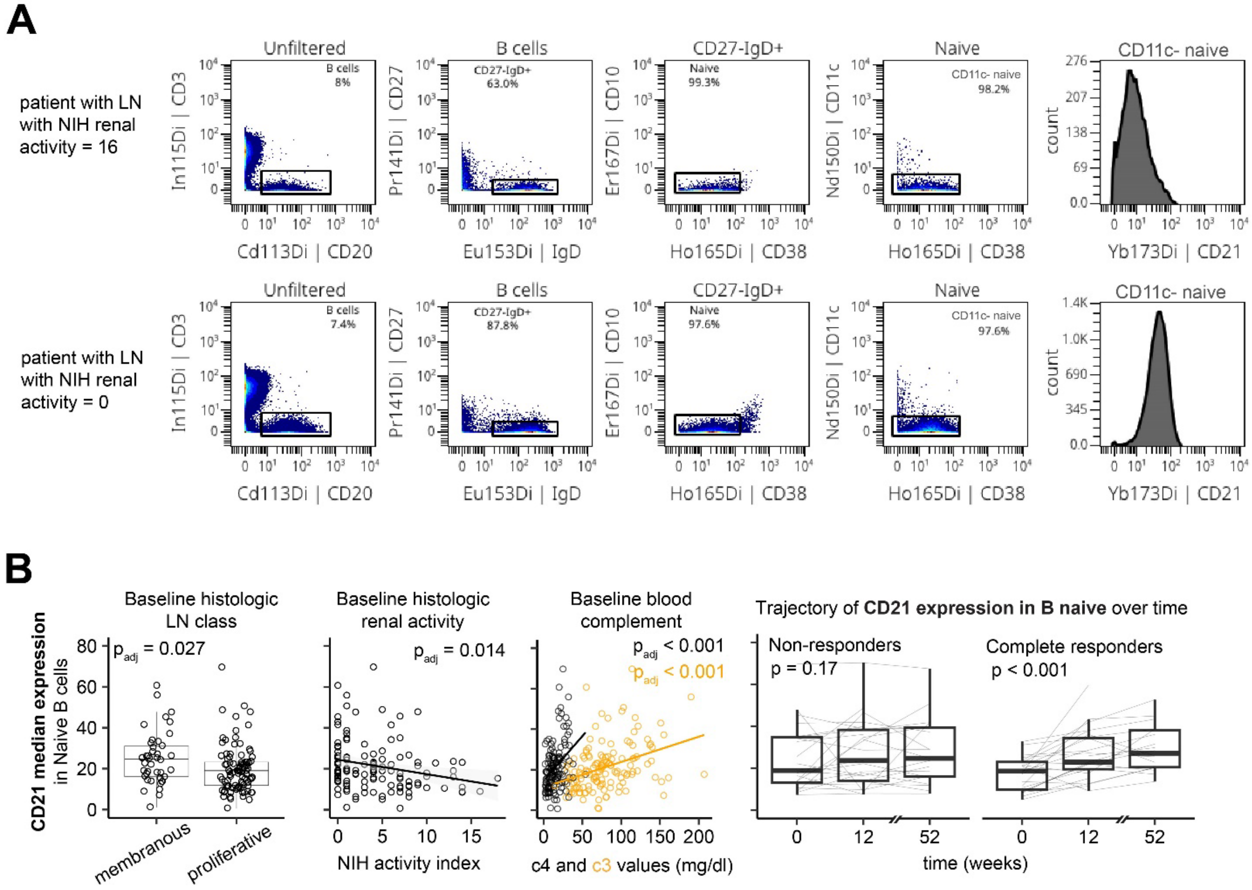

Supplemental Figure 5. Naïve B cells shift their phenotype towards a low expressing CD19 and CD21 profile in active and proliferative LN patients. (**A**) Representative examples of CD21 expression in manually gated naïve B cells in a patient with LN with high vs low NIH renal activity. (**B**) Median CD21 expression amongst naïve B cells association with histological characteristics, complement values (C3 and C4) and longitudinal change over time. Statistical significances were determined either using Wilcoxon sum-rank test (LN class), Spearman's rho correlation (NIH activity index and complement) or a mixed effect model with patient as a random effect (changes over time).

Supplemental Figure 6

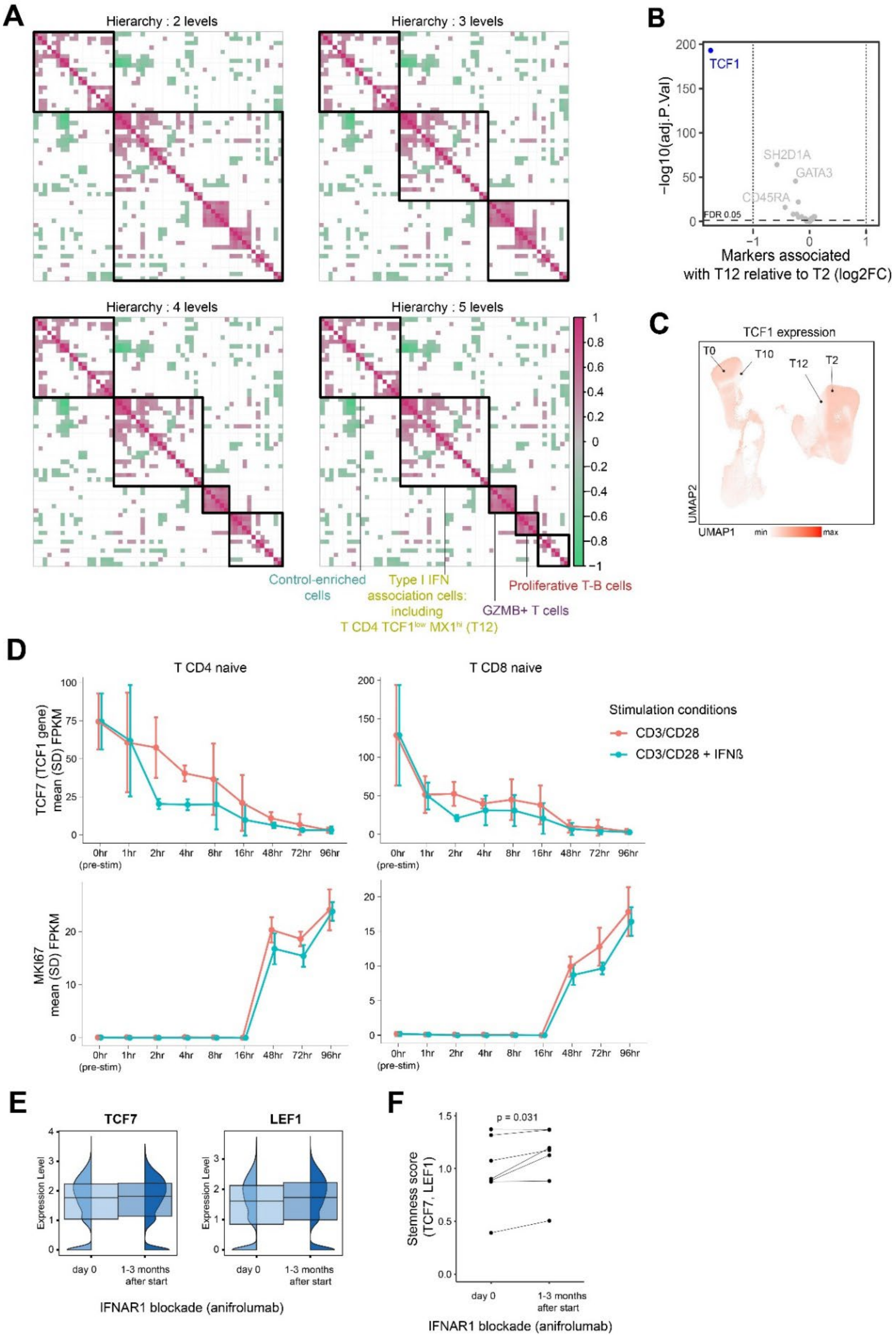

Supplemental Figure 6. Hierarchical clustering of co-correlated cells identifies cell subsets strongly associated with type I interferon signaling. (A) Heatmap of correlations between 55 immune cell subsets, organized by hierarchical clustering. Black boxes indicate the first five levels of hierarchy, with key co-correlating cell sets labeled by their defining characteristics. (**B**) Differential expression of T-panel markers between T-cell subsets T2 and T12, determined using the limma package. (**C**) UMAP of TCF1 expression in T cells. (**D**) Changes in TCF7 (TCF1 gene) and MKI67 (Ki67 gene) expression in healthy naïve CD4 and CD8 T cells stimulated with or without interferon-β, using published bulk RNA-seq dataset. (**E-F**) Single-cell representation of TCF7 and LEF1 gene expression (E) and median stemness score (combining TCF7 and LEF1) (F) in T cells from 7 patients with lupus erythematosus before (day 0) and after IFNAR1 blockade treatment (1–3 months post-anifrolumab).

Supplemental Figure 7

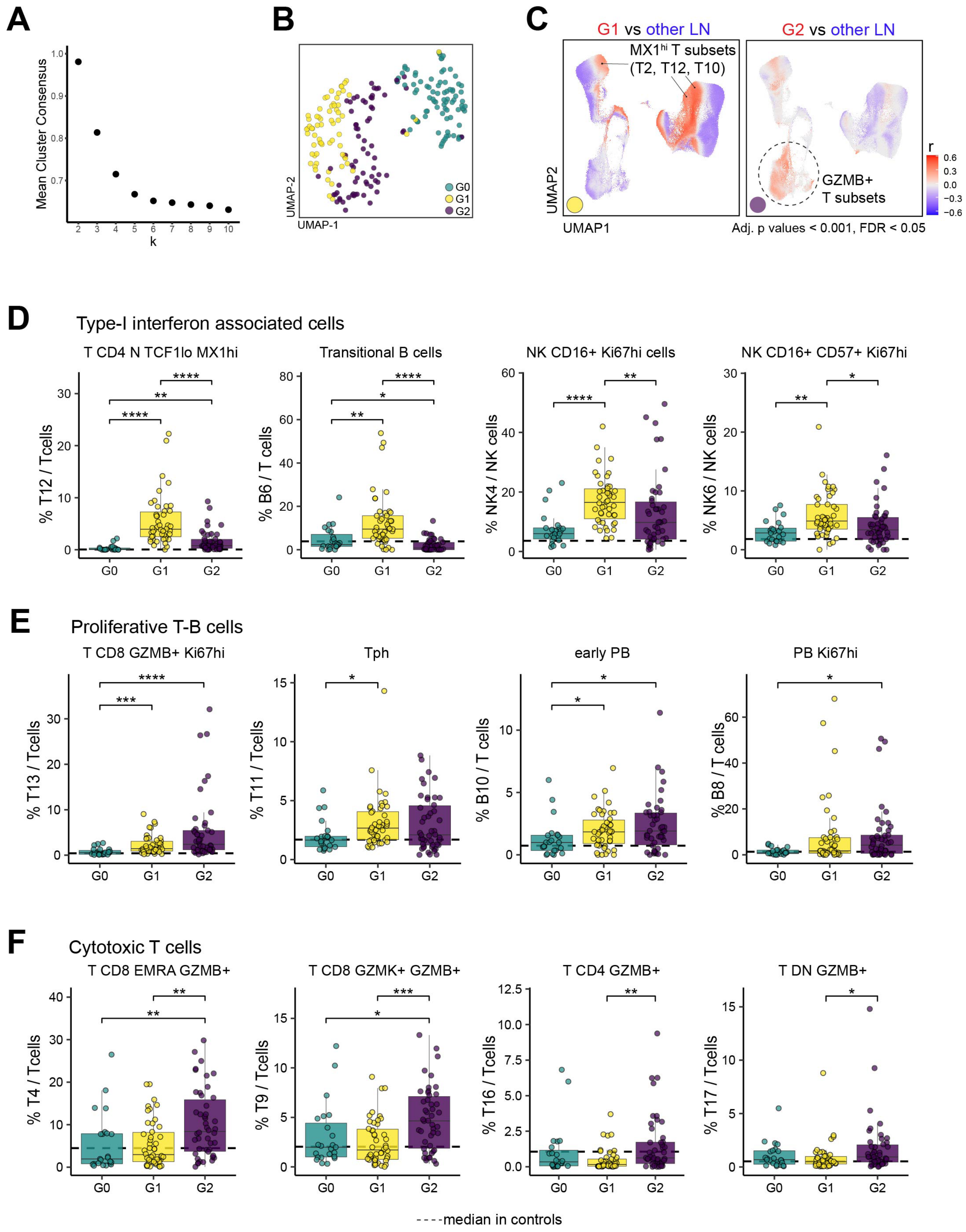

Supplemental Figure 7. Circulating immune cell subsets characterizing three LN groups. (A) Stability of sample membership to clusters depending on the number of clusters by repeating 1000 K-means clustering on a resampled dataset without replacement. (**B**) UMAP distribution of all samples included in this study (n=267) based on the proportion of 55 immune cell subsets. (**C**) Cell neighborhood associations with the K-means defined groups G1 and G2 relative to the other groups. (**D-F**) Comparison of selected cell subsets between the three K-means defined groups of LN patients at baseline (23 G0, 46 G1 and 46 G2). Statistical significance was determined by Kruskal-Wallis test followed by Dunn's multiple comparisons.

Supplemental Figure 8.

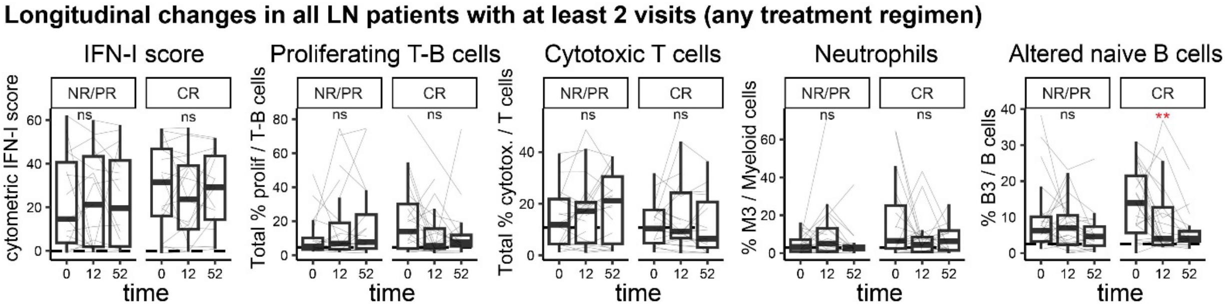

Supplemental Figure 8. Longitudinal changes in immune cell signatures stratified by response status (NR/PR = none/partial, CR = complete), including all patients with LN with at least 2 timepoints. Statistical significance was determined using a mixed effect model including patient as a random effect. *p<0.05, **p<0.01, ***p<0.001.

Supplemental Figure 9

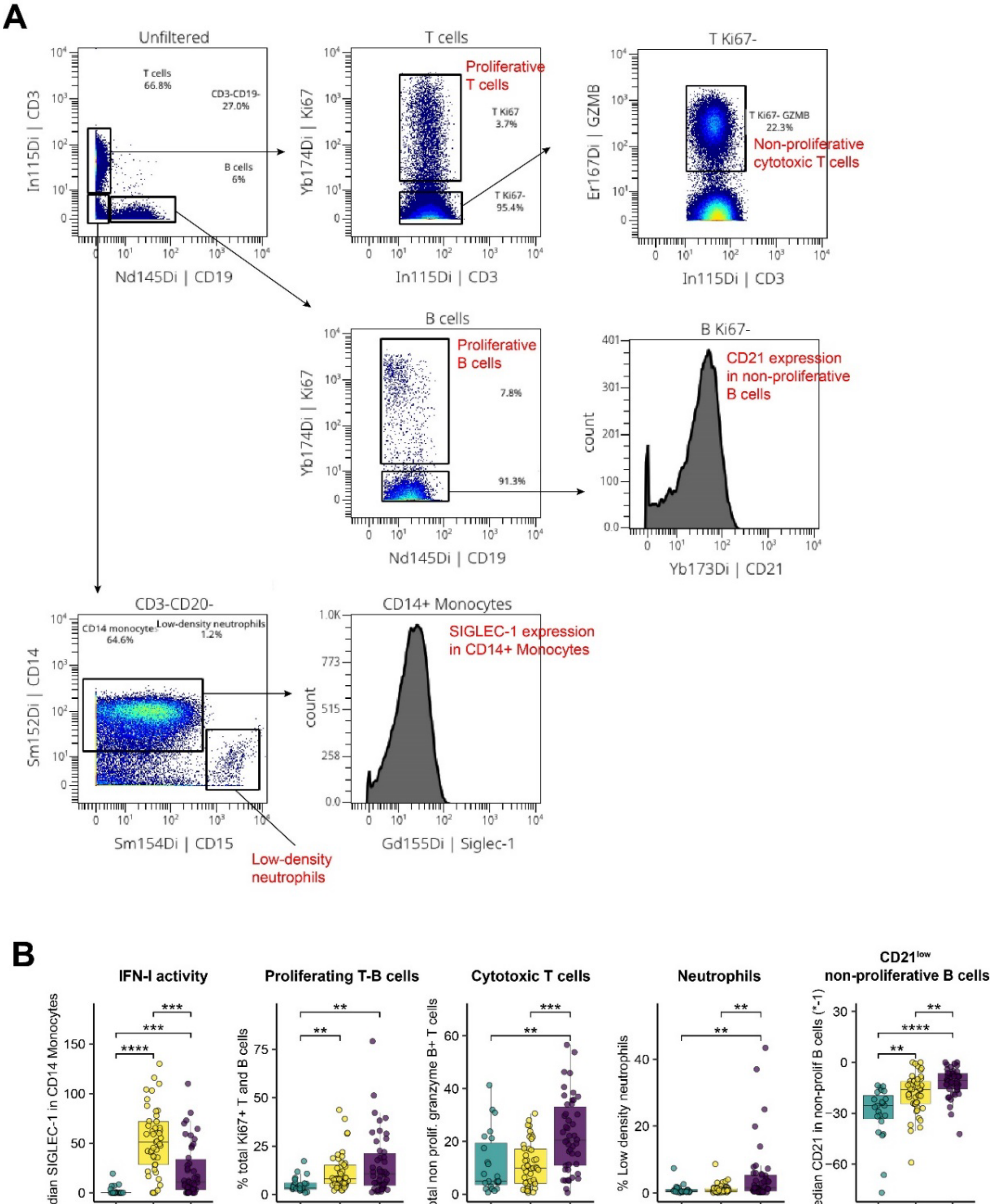

Supplemental Figure 9. Simplified cellular immunophenotype. (**A**) Proposed gating strategy to obtain simplified cellular immunophenotypes. (**B**) Comparison of baseline cellular immunophenotypes between blood-defined LN groups (G0 = 23, G1 = 46, G2 = 46).

Supplemental Figure 10

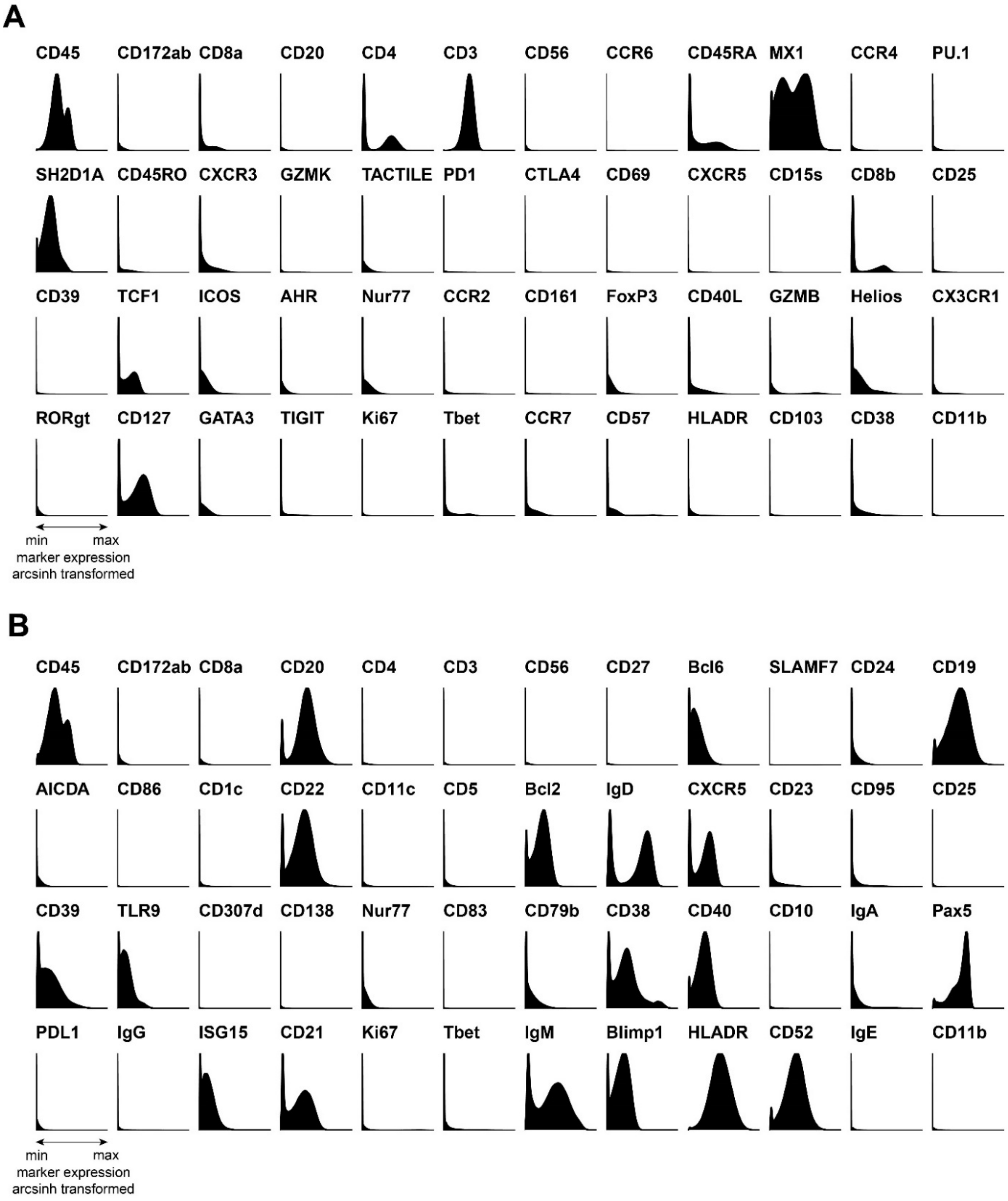

Supplemental Figure 10. Histograms of T and B-cell mass cytometry makers. (**A-B**) Includes all markers from the T-panel expressed by T cells (A) and B-panel expressed by B cells (B)**.**

Supplemental Figure 11

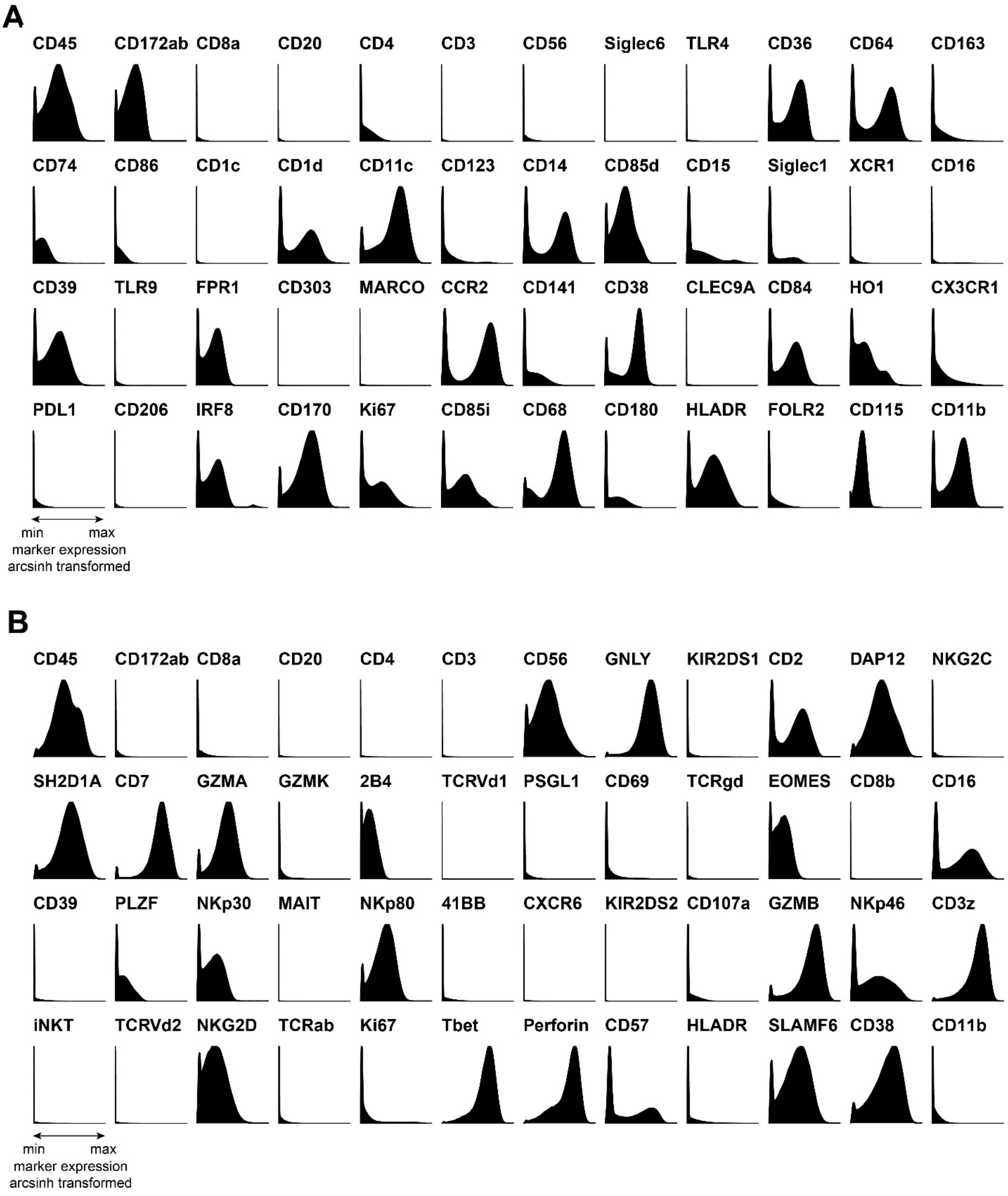

Supplemental Figure 11. Histograms of myeloid and NK-cell mass cytometry makers. (**A-B**) Includes all markers from the myeloid-panel expressed by myeloid cells (A) and NK-panel expressed by NK cells (B).

Supplemental Figure 12

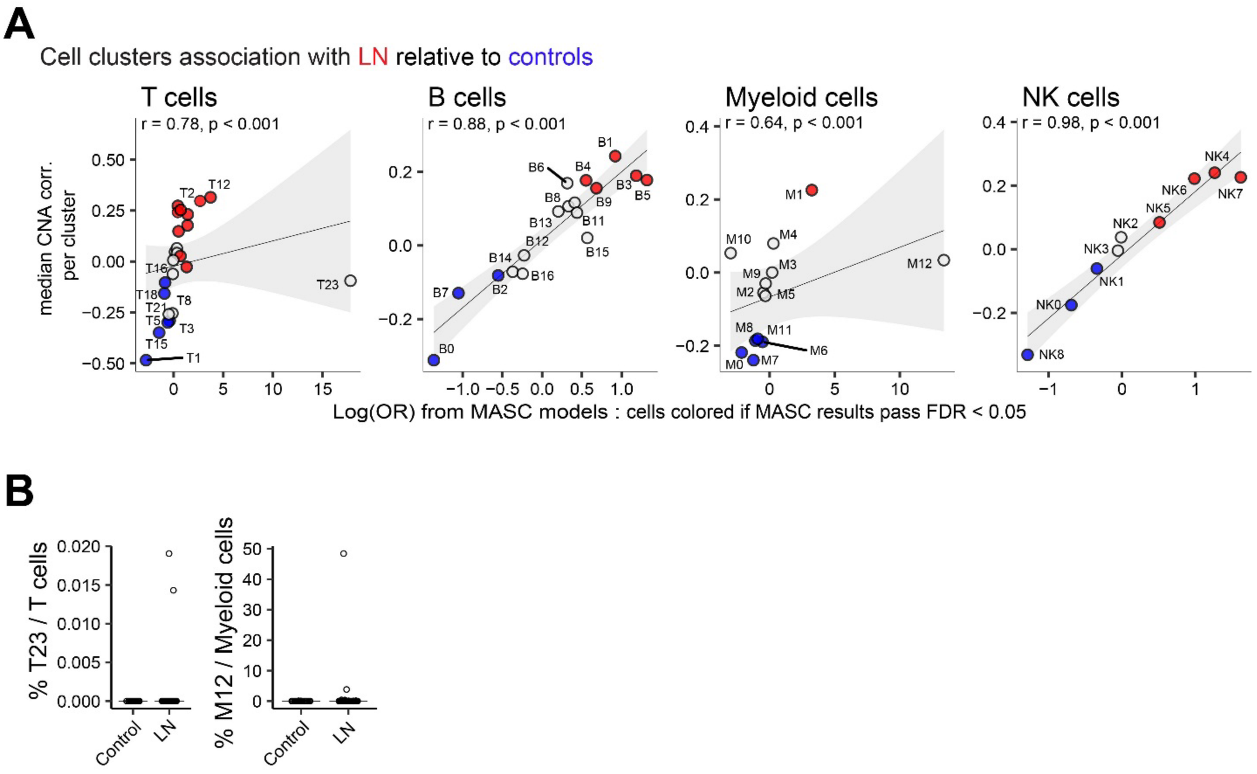

Supplemental Figure 12. Comparison of CNA and MASC analysis. (**A**) Identification of cell clusters associated with LN (max n=140 in the B panel and min 116 in the NK panel) relative to controls (max n=40 in all panels except for n=39 in the NK panel), using two different approaches : covarying neighborhood analysis (CNA) and a single-cell mixed-effect model (MASC). Plots represent the correlation between the two models using Spearman's rho. (**B**) Example of two cellular cluster outliers identified as outliers in figure A.
